# Neckband-type earphone for continuous monitoring of cardiovascular symptoms via self-powered box knot pulse-wave sensor

**DOI:** 10.1038/s44385-025-00007-z

**Published:** 2025-02-12

**Authors:** Tae-Ho Kim, Dominic Jaworski, Rakesh Sethi, Elise Huisman, Kam Fung, Edward J. Park

**Affiliations:** 1 Biomechatronic Systems Laboratory, School of Mechatronic Systems Engineering, Simon Fraser University, Surrey, BC Canada; 2 WearTech Labs, Simon Fraser University, Surrey, BC Canada; 3 Research & Technology and Open Innovation, Acute Care & Monitoring, Medtronic, Boulder, CO USA

**Keywords:** Biotechnology, Computational biology and bioinformatics, Physiology, Cardiology, Signs and symptoms

## Abstract

Blood pressure (BP) assessment is one of the essential vital signs in the clinical field because of its significant relation with various chronic diseases. For effective continuous BP monitoring at home, the system should be portable, user-friendly, and comfortable for the patient, ensuring convenience during continuous data collection. Here, a wearable neckband-type earphone for continuous monitoring of cardiovascular symptoms and BP in a non-invasive and wireless manner via a Self-powered Box Knot Pulsewave sensor (SBKP) has been reported. The multi-stacked architecture of the SBKP, inspired by the knotting craft, holds a sensitivity and resolution of 38.17 mV Pa^−1^, and 0.006 kPa, respectively, which allows for the measurement of the human pulse waves from the cheek, neck, and wrist. Additionally, its low energy consumption sensor, achieved by the triboelectric mechanism, enables us to develop additional user-convenient auxiliary care systems: continuous BP monitoring with potential music therapy.

## Introduction

Currently, the ratio of medical doctors to patients in hospitals in British Columbia, Canada, stands at 2.47 per 1000 individuals^1^. This statistic reflects the intense workload shouldered by healthcare professionals. Additionally, the shortage of out-of-clinic care services, such as home-based continuous vital sign monitoring, continues to add to the strain on the healthcare system. Moreover, the current auxiliary systems designed to support both the patients and clinicians are often selective and complex, making them challenging to manage, resulting in substantial healthcare costs^2,3^. Consequently, wearable electronics for blood pressure (BP) assessment have emerged as a promising solution among auxiliary system technologies, as BP monitoring is a critical vital sign used to assess patient conditions in clinical settings^4,5^.

High BP, known as hypertension, is closely associated with various chronic diseases and has a direct impact on patients’ health^6^. Furthermore, the mortality rate related to BP issues has been steadily increasing, reflecting the urgent need for active BP monitoring systems^7^. Developing an active, continuous BP care system is particularly difficult with conventional methods such as cuff-type sphygmomanometers, as these require over 30 s of waiting time for airbag inflation and deflation to obtain BP values^8^. Additionally, its portability remains a concern for outpatient clinic services, as the process of donning and doffing the cuff is inconvenient for patients and the equipment can be bulky (although smaller commercial devices are available). Therefore, there is a growing need for cuffless-type BP measurement systems^9^.

Electro-mechanical coupling of the heart results in blood flow into the arteries, distributing oxygenated blood throughout the body. This process affects the blood velocity from the central to peripheral arteries, defined as a systemic pressure wave. Such a pressure wave expands the path of the blood flow and moves faster than the flow itself^10^. Based on these heart behaviors, BP changes are caused by two key factors: (i) heart rate directly related to the ventricular contraction and the pressure resulting in blood flow in the arteries during each beat^11^ and (ii) the elasticity of the arteries, which allows for the expansion of the blood flow path^12^. These heart-artery behaviors can be measured using various types of physiological sensors, providing clues for developing non-invasive BP measurement methods, known as cuffless BP measurement^13,14^.

The cuffless BP measurement requires the use of two or more paired cardiac-relevant sensors. By analyzing the paired sensors, the interval times between two sensor signal peaks, called pulse transit time (PTT), can be calculated^15^. Based on this time, BP can be estimated after establishing the relationship between PTT and BP obtained from the paired sensors and cuff-type sphygmomanometer, respectively^16^. Such methods require only simple contact of the paired sensing units with the human skin. This presents a great advantage in allowing continuous measurement of BP outside of a clinical setting, using mobile or wearable devices. For example, wearable BP assessment systems using paired electrocardiogram (ECG) and photoplethysmography (PPG) sensors, equipped with bendable or stretchable light-emitting diodes or electrodes, have been reported^17^. Additionally, robot-assisted BP assessment and other healthcare systems have been demonstrated, incorporating such wearable devices with humanoid robots^18,19^. Also, various sensor combinations are available for cuffless BP measurements, including pressure-pressure^20,21^, ECG-vibration^22^, and ECG-ultrasound paired sensors^23^. These cuffless BP measurement technologies are promising for continuous clinical BP monitoring and outpatient services due to their adaptability to wearable devices^20–24^.

For effective continuous BP monitoring, an ideal system is expected to be portable, user-friendly, and comfortable for the patient, ensuring convenience during continuous data collection. However, the reported cuffless-type BP measurement systems still require further enhancements to provide usefulness in home settings. Despite various prospective studies over the past decade aiming to develop wearable sensors, including conjugate polymer-based materials for photoplethysmography^25^, ultrasound wall-tracking methods^26^, carbon nanotubes^27,28^, graphene^29^, piezoelectric materials^21,30^, metal nanowires^31^, and conductive fibers^32^ for mechanical ballistocardiography−most of these studies have been conducted with incomplete prototypes. Such an approach fails to fully address the practical conditions under which these devices would operate^33^. Typically, the sensors are simply affixed to various body parts, operating in isolation rather than as part of an integrated and complete wearable system such as other portable consumer electronics like smartwatches or neckband-type earphones (e.g., Sony WI-1000XM2 or LG HBS-510 Wireless Bluetooth Headset). A promising wearable device should prioritize a compact system design for overall functionality and effortless portability.

Among the sensor mechanisms used to measure human pulse waves for one of the BP sensor pairs, the triboelectric mechanism is a promising candidate. This mechanism, which operates via static electricity, is commonly observable in everyday items such as plastic file holders or clothing, and even from their waste materials. It generates high-voltage output with very low current, implying it could detect human pulse waves through distinctive voltage variations^34^. The triboelectric sensing concept, as a so-called self-powered system, is also highly attractive to fully portable devices, as it alleviates energy consumption issues^35^.

Since the triboelectric mechanism was first reported^36^, a wide range of research has been conducted to improve (increase) voltage or current for better power outputs^37^. From a material perspective, there have been meaningful attempts to modify the material surfaces to enhance the charge affinities of triboelectric materials, even employing nano-^38^ and micro-scale particles^39^ or wires^40^. From a structural perspective, innovative triboelectric sensor designs with enlarged effective contact areas have been developed, inspired by conventional architectural approaches or arts. One well-known approach involves using paper art-derived engineering structures, such as origami^35^ and kirigami^36^, to create multi-layered or stacked architectures that improve the effective contact area and often significantly increase the power output of triboelectric mechanism. Furthermore, these designs enable common materials to generate comparable triboelectric sensor performance comparable to that of conventional materials^41–44^.

In terms of multi-stacked structures, knotting provides an excellent source of inspiration for the triboelectric mechanism. A knot is an intentionally convoluted structure in cordage^45,46^, Among these, the box knot, a traditional Korean knot, has a multi-stacked architecture, as shown in Supplementary Fig. 1. Its uniformly stacked patterns imply potential improvement in the effective contact areas between two paired triboelectric materials. Furthermore, this architectural perspective opens new possibilities for achieving superior sensor performance through various material innovations. Thus, we performed the studies under a material-independent view, and polytetrafluoroethylene (PTFE)—polyethylene terephthalate (PET) paired materials were selected as representative materials for our approaches.

This paper presents the design of a compact neckband-type earphone prototype developed for practical cuffless-type BP measurement systems. This neckband primarily focuses on new hardware development that benefits home users. Thus, our BP monitoring neckband-type earphone includes wireless monitoring systems and Bluetooth pairing with personal smartphones for music listening or phone calls. By retaining the original functions of the earphone, we aimed to pursue further user-convenient auxiliary care systems such as potential music therapy for cardiovascular health or personal communication, beyond simply monitoring vital signals. The self-powered box knot pulse-wave sensor (SBKP) for human pulse wave detection has been integrated into the neckband earphone. Its pulse wave-detecting performance was verified using a conventional PPG sensor, which was also included in the device set for comparison purposes. After calibration processes^15,16^ with the proposed ECG-SBKP pair, the BP-monitoring neckband-type earphone was demonstrated in two ways: (i) continuous BP monitoring of human subjects on different posture conditions and (ii) continuous BP variation of the human participant at two different music listening sessions.

## Results

### Earable self-powered box knot cuffless BP sensor

**Fig. 1:**
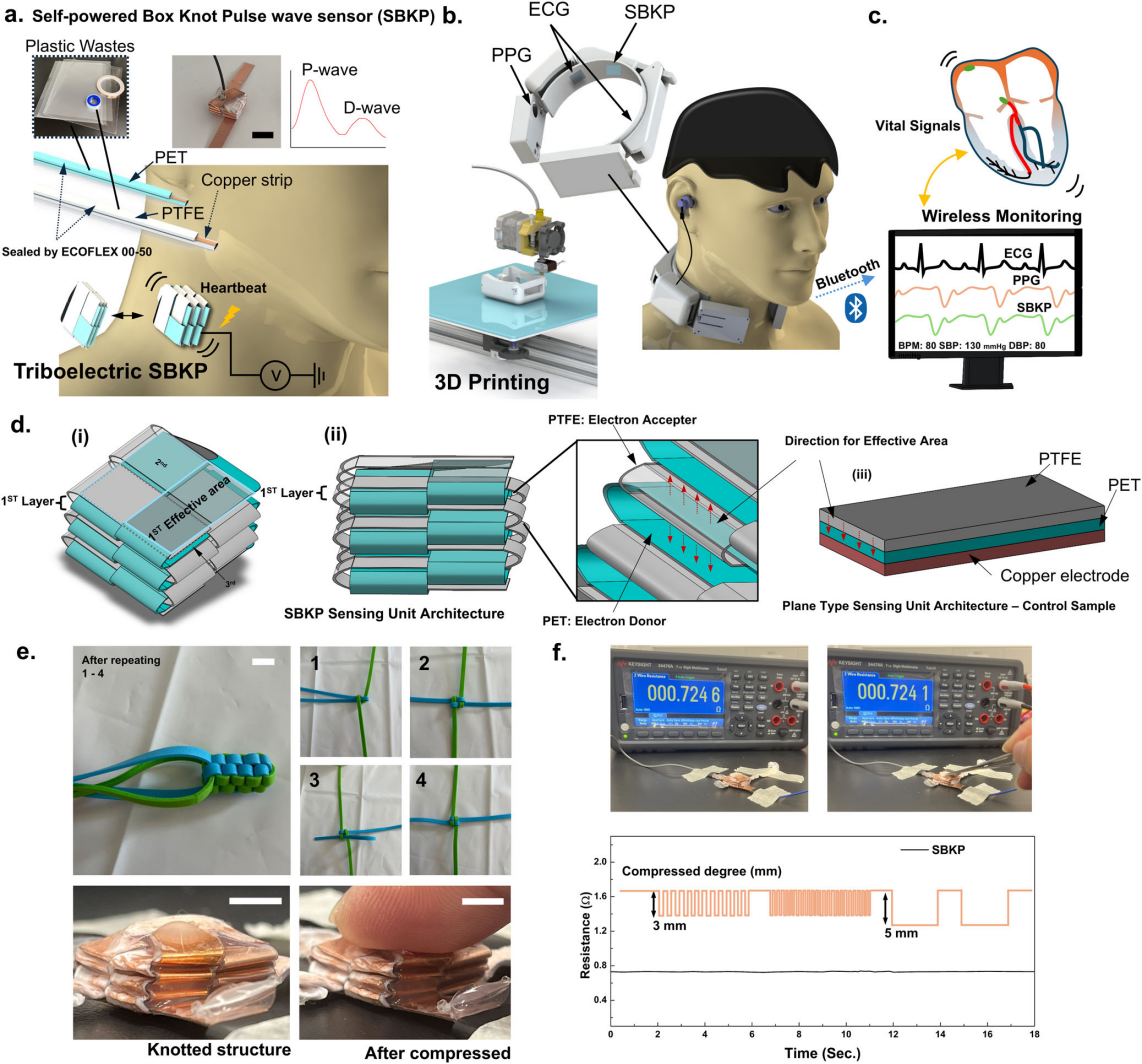
Earable self-powered box knot cuffless BP sensor. **a** Schematics of self-powered box knot wave sensor (SBKP) fabricated using PET and PTFE plastic wastes, and its actual photo. **b** The SBKP has been integrated with ECG and PPG sensors and incorporated into the 3D-printed neckband-type earphone to develop a wearable device for continuous diagnosis of cardiovascular symptoms and blood pressure. **c** This device includes a wireless monitoring system through the Bluetooth connection with the personal computer. **d** Schematics of the SBKP architecture: (i) top view, (ii) side view, and (iii) a plane-type sensing unit architecture as the control sample. **e** Photos to represent knotting methods to generate box knot structure, and actual SBKP sample before and after compressed. **f** Stable resistance profiles of the SBKP copper strip at multiple compression with 3 and 5 mm compressed degrees. Scale Bars: 7.5 mm

Figure 1 shows schematic overviews of the proposed cuffless, self-powered box knot BP sensing neckband earphone. Regarding the self-powered box knot, two different strip materials with different charge affinities are knotted to form a box-shaped architecture. This knotting method creates uniformly paired, multi-stacked, three-dimensional structures, as shown in Fig. 1a. The sensor measures mechanical heartbeats on the human neck to generate pulse wave signals. The triboelectric mechanism generates a voltage output when the two knotted strips move closer together due to deformation from the mechanical pulse beat. Also, it generates voltage outputs through alternating current (AC), requiring the use of a bridge amplifier. For our triboelectric sensor, we employed a stand-alone (or single-electrode mode) type sensor^34,47^. Only the PET layer, which has a positive charge affinity, is integrated into the conductive wire to deliver the voltage signals. The PTFE layer, due to its fluorine pendant chains, has a much higher electronegativity than PET and acts as the negatively charged layer. When the PET and PTFE layers are paired, the charge difference between them generates a current flow as they approach each other. It is important to note that our studies primarily focus on architectural knotting approaches from a material-independent perspective, with paired PET-PTFE used as a representative model. However, as the layers move apart, the current flow is generated in the reverse direction, resulting in the triboelectric sensor generating AC by default. After fabricating the triboelectric sensor, it was integrated into a 3D-printed neckband-type earphone (Fig. 1b) along with ECG-PPG paired sensors. With additional functional features, such as wireless communication with other devices, the complete device was developed as shown in Fig. 1c. Our developed wearable device can measure three different signals simultaneously and can be monitored wirelessly through personal computers. Additionally, the device can be connected to a personal smartphone for its primary function as an earphone, allowing real-time BP monitoring while participants listen to music. Our BP monitoring system also offers the potential for developing further user-convenient auxiliary care systems, such as music therapy, beyond simple BP monitoring. Therefore, continuous BP monitoring under different music conditions has also been demonstrated in this paper.

Our triboelectric sensor has been generated using architectural perspectives. Fundamentally, it is known that a larger effective contact area between two different triboelectric materials induces better energy-harvesting performance of the triboelectric mechanism^31^. Therefore, strategies to design the stacked spatial architected structure, specifically knotting methods, have been considered. Knotting has been widely used to maximize the holding force of rope, and naturally, the shape of multi-stacked bundles is considered to disperse the normal force (or drag). However, such stacked bundles can also inspire improvements in triboelectric performance. Herein, the box knot design has been introduced, featuring a uniformly stacked structure in a box shape, as shown in Fig. 1d(i). Such box knots have been used as decoration for key rings or backpacks, as shown in Supplementary Fig. 1. Thanks to its multi-stacked structure in Fig. 1d(ii), the total effective area between interlaced strips can significantly increase while the top view area made by the first knotting layer remains small. After repeated knotting three times, 675 mm^2^ of the apparent area has been obtained from the knot, but its plane area in the top view is 15 × 15 mm^2^. Additionally, a plane-type triboelectric sensing unit with a 27 × 25 mm^2^ effective area has been prepared as a control sensing unit, illustrated in Fig. 1d(iii) as a control sample. For the box knot sensing unit, we expected comparable performance in pulse wave sensing with less contact area between human skin and sensing units compared to the control sensing unit, contributing to a more compact overall device setting. Moreover, the second layer of the box knot structure provides an additional effective (or contact) area to set the contact pairs in up and down directions as shown in the inset of Fig. 1d(ii), unlike the control sensing unit. Therefore, we assumed a triboelectric voltage output ratio between the box knot sensing unit and its control sensing unit through Eq. (1)^48^,

1 $$V=-\frac{{{\rm{Q}}}_{t}}{{{\rm{A}}}_{a}\varepsilon }\left({d}_{t}+D\right)+\frac{{\rm{\sigma }}D}{\varepsilon }$$

where Q_t_ is the amount of tribo-charges formed on the triboelectric (or dielectric) material surface, defined as ${Q}_{t}={A}_{e}({\widehat{{\rm{W}}}}_{1}-\,{\widehat{{\rm{W}}}}_{2})$. $\widehat{{\rm{W}}}$ is the work function of the materials depending on the applied force and separation between two different triboelectric layers, PTFE and PET in our case. A_e_ and A_a_ are the effective and apparent areas between them. ε is the permittivity of a gap between the layers. σ, d_t,_ and D are the surface charge density, thickness of two triboelectric layers, and the gap distance, respectively. The ratio of the estimated voltage output for box knot (V_b_) and (V_p_) control sensing units is obtained using Eq. (2),

2 $$\frac{{V}_{b}}{{V}_{p}}=\frac{-\frac{{{\rm{Q}}}_{{t}_{b}}}{{{\rm{A}}}_{{a}_{b}}\varepsilon }\left({d}_{{t}_{b}}+{D}_{b}\right)+\,\frac{{{\rm{\sigma }}}_{b}{D}_{b}}{\varepsilon }}{-\frac{{{\rm{Q}}}_{{t}_{p}}}{{{\rm{A}}}_{{a}_{p}}\varepsilon }\left({d}_{{t}_{p}}+{D}_{p}\right)+\,\frac{{{\rm{\sigma }}}_{p}{D}_{p}}{\varepsilon }}=\frac{{{\rm{Q}}}_{{t}_{b}}}{{{\rm{Q}}}_{{t}_{p}}}$$

Both sensing units were fabricated using the same materials, gap between layers, and apparent area. Therefore, the ratio can be correlated to the ratio of their effective layers. Also, only the second layer of the box knot sensing unit was considered to have an additional effective area (Fig. 1d(ii)). From Eq. (2), the box knot sensing unit was estimated to have a 33% higher voltage output than the control sensing unit. However, this estimation is only valid when the force is fully transferred from the bottom to the top of the box knot sensing unit uniformly, which may raise concerns about potential overestimation. Therefore, we conducted actual experiments to evaluate the box knot’s performance relative to the control sensing unit.

Moreover, after generating the box knot structure shown in Fig. 1e, potential defects in the conductive copper strips may arise, as the knotted structure may involve fully folded sections. Thus, the resistance of each strip was inspected. The resistance of the strip was 0.731 ± 0.003 Ω, and no significant changes in the resistance were observed during the repeated compressions of the sample, with 3 mm and 5 mm compressed degrees at different compressive frequencies (Fig. 1f). These results suggest that the knotting methods used to generate the box knot structure in this study create a bending structure rather than fully folded structures, which contributes to the electrically stable conductive strip after forming a multi-stacked structure. In terms of the long-term durability of the SBKP structure, we demonstrated its potential in the next section.

### Performance of the triboelectric box knot

**Fig. 2:**
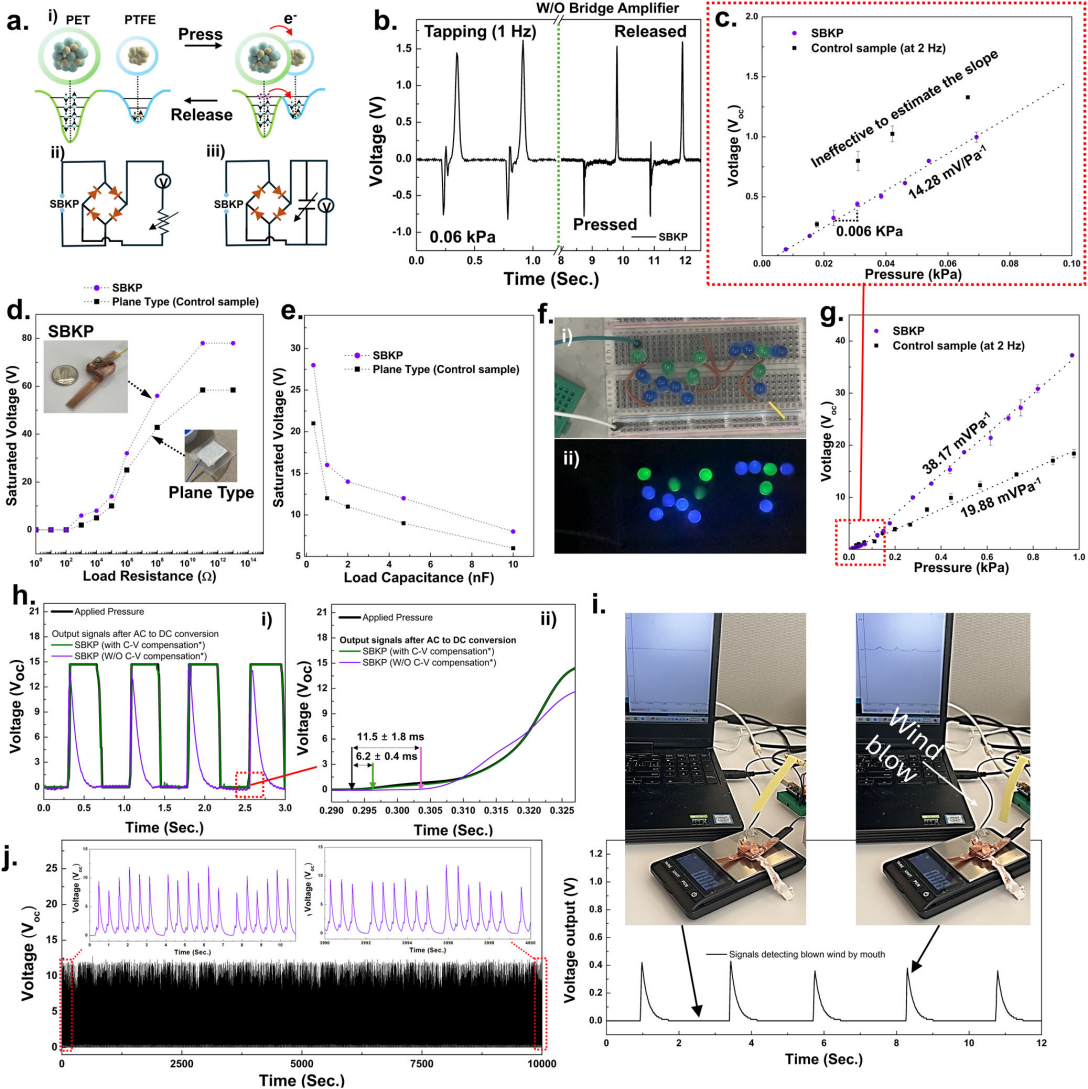
Performance of the triboelectric box knot. **a** Schematics of triboelectric mechanism (i), and equivalent circuits for the experimental testing setup to evaluate the voltage outputs depending on load resistances (ii) and capacitances (iii). **b** The charge/discharge curves obtained from SBKP reflect the AC output generated from the triboelectric mechanism at its pristine condition. **c** Representative graph to show the resolution of the SBKP and control samples at 0.0 to 0.1 kPa range. Voltage outputs of SBKP and plane-type sensing units (control sensor unit) at different (**d**) load resistances and (**e**) capacitances. **f** Photos of an LED module integrated with SBKP before (i) and during (ii) tapping its sensing unit. **g** The continuous electrical output of the SBKP and control samples in response to the continuously applied external pressure in a measurement cycle. **h** Response time characterization of the SBKP. **i** Signals of the SBKP detecting blown wind by mouth. **j** Sensor mechanical robustness test.

The performance of the triboelectric box knot has been characterized. While the C-F bonds within PTFE are highly polar due to the high electronegativity of fluorine, the polymer as a whole does not exhibit a net dipole moment and is thus non-polar. This non-polarity results from the symmetric arrangement of these polar bonds, not from a low electronegativity of the molecule itself. Thus, as illustrated in Fig. 2a(i), the fluorine atoms in PTFE have a strong electron-withdrawing effect, attracting electron density towards themselves and creating regions of high electron density around the fluorine atoms. Although this electron-withdrawing effect is known to stabilize the PTFE molecule by lowering its overall energy, it also enables PTFE to act as an electron acceptor. Moreover, it is known that PET has a much lower triboelectric charge density (TECD) compared to PTFE, with values of −1.09 μC/m^2^ and −27.5 μC/m^2^ against a copper board, respectively^49^. Therefore, the PET layer was selected as an electron donor^49^.

A customized testing system was developed using a vibrating motor triggered by a function generator. As shown in Fig. 2a(ii), (iii), two different equivalent circuits were employed to evaluate the voltage output based on the load resistance and capacitance^50,51^. First, the AC output generated by the SBKP was observed in Supplementary Fig. 2 using the circuit without a bridge amplifier, as the voltage output is generated twice: when the two triboelectrically paired layers approach and detach. Around 78 V of open-circuit voltage (V_oc_) was measured, with different current directions noted as charge and discharge in the graph (The V_oc_ at the 0.06 kPa pressurized condition is presented in Fig. 2b). Also, two different periodic impacts, at 2 Hz and 5 Hz, were applied to the SBKP, as shown in Supplementary Fig. 2a, demonstrating that the sensor maintains consistent output under different frequencies. Higher frequencies were not considered, as the sensor is primarily designed to detect human pulse waves, which typically have frequencies below 3 Hz or 5 Hz for stable percussion (P-) and diastolic (D-) wave detection related to systolic and diastolic blood pressure, as well as heart rate^52^.

Another important factor in measuring pulse waves is sensor resolution, as pulse waves are generated with subtle mechanical forces. Thus, we investigated the resolution of the SBKP and control samples at a low-pressure range Fig. 2c. We applied tapping pressure five times across a range of 0 to 0.1 kPa, increasing in 0.005 kPa intervals, while simultaneously collecting the V_oc_. Also, we defined the resolution as the interval of applied pressure that produces a distinctive variation in V_oc_ and considered an area effective if it exhibited noticeable V_oc_ changes across the pressure range and intervals. Although the control sample appeared to have higher sensitivity at this range, it was difficult to define its sensitivity due to multiple ineffective areas that complicated the matching points of signal output with applied pressure. The control sample’s resolution was assumed to be around 0.018 kPa, even though this was not clear because of the ineffective areas. In contrast, the SBKP showed a resolution of 0.006 kPa, with uniform, effectively measured plots.

The voltage output at different load resistances is an important feature for energy harvesting^51,53^. Thus, the voltage output of the SBKP and the control sample sensor was measured across a load resistance range of 10 ~ 10^13^ Ω. The saturated output voltages of 78.2 and 58.4 V were obtained at a load resistance of 10^11^ Ω for the SBKP and the control sample sensor, respectively (Fig. 2d), with the SBKP showing a 26% higher output than the control sample, but lower than the estimation using possible effective areas in the previous section. The corresponding power outputs were also calculated, with maximum values of 0.42 for the SBKP and 0.18 W/m^2^ for the control sample sensor.

Furthermore, the saturated voltages at different load capacitances were measured (Fig. 2e) to calculate the energy (E) stored in the capacitor using the equation: *E* = *CU*^*2*^*/2*, where *C* is the capacitance, *U* is the saturated voltages^54^. When the SBKP was subjected to continuous periodic impacts at a frequency of 2 Hz, the voltage across the capacitor was measured until it saturated after 10 s. Maximum energy of 338.4 nJ was obtained from the SBKP at a 4.7 nF load capacitance, which is 44% higher than that of the plane-type control sensor. For detailed power output and energy storage across other load resistances and capacitances, see Supplementary Figs. 2b, c.

Based on these results, it can be concluded that the improved effective areas caused by the multi-stacked structure of the SBKP contributed to its superior performance as a triboelectric sensor. As shown in Fig. 2f and Supplementary Movie 1, the SBKP was able to light up over 15 LEDs that were integrated in series, demonstrating its capability as a self-powered mechanism for sensor applications. Before actually applying and developing a human pulse wave sensing device with the SBKP, additional performance evaluations were conducted. Figure 2g shows the continuous electrical output of the SBKP in response to the continuously applied external pressure in a measurement cycle. At a constant frequency of 2 Hz, the voltage output of the SBKP was measured while gradually increasing the applied pressure. The voltage–pressure curves of the SBKP and control samples exhibit hysteresis, with errors of 3.5% and 5.8%, respectively. In the pressure region of ~1 kPa, the voltage increases linearly until an applied pressure of 0.44 kPa is reached. A pressure sensitivity of 38.17 mV Pa^−1^ was calculated from the slope of the linearly fitted relationship between V_oc_ and applied pressure (sensitivity = V_oc_/applied pressure). In contrast, the control sample exhibited a sensitivity of 19.88 mV Pa^−1^.

The response time (Fig. 2h), long-term sensing (Fig. 2i), and representative sensing performance (Fig. 2j) of the SBKP are also presented. Under multiple applied forces of 0.4 kPa, the SBKP showed good correspondence with the applied force, as seen in Fig. 2h (i). From the edge of the response profile in Fig. 2h(ii), the response time of the SBKP was calculated. It is important to note that voltage is only actively generated during the separating or pressurizing phases, when the paired triboelectric layers are either being pressed together or separated, resulting in opposite current directions^55^. Thus, only the response time for the pressurized phase was measured after employing a bridge amplifier to convert AC to DC voltage in the equivalent test, yielding a response time of 11.5 ± 1.8 ms^51^. After applying capacitance-to-voltage (C- to-V) conversion compensation to the SBKP, the response time dramatically improved to 6.2 ± 0.4 ms, with voltage retention until the sensor was released^20,56^. For details on the C-to-V compensation circuit^57^, please see Supplementary Fig. 3. Moreover, we tested whether the sensor could even detect soft wind blows by mouth as a representative demonstration of its performance (Fig. 2i). The voltage signal caused by a wind blow was around 0.42 V, corresponding to a pressure of 0.025 kPa (see Supplementary Movie 2 for further details). Finally, the sensor exhibited stable sensing performance over 20,000 cycles of force application (Fig. 2j). Based on the above evaluations, the SBKP sensor shows significant potential for developing a pulse wave sensing device.

### Self-powered box knot pulse-wave sensor device

**Fig. 3:**
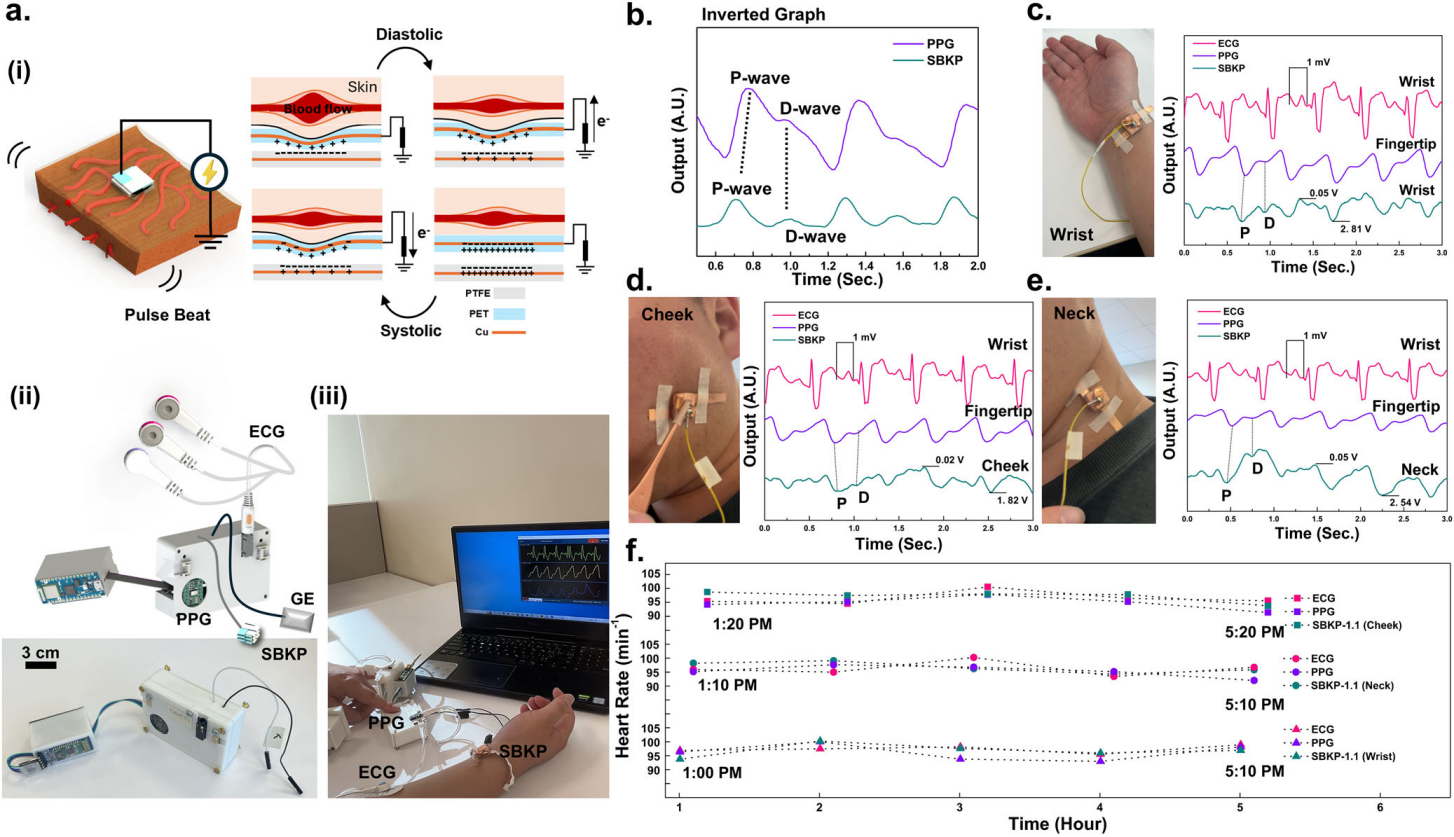
Self-powered box knot pulse-wave sensor device. **a** Schematics to illustrate the pulse wave detecting mechanisms of SBKP (i), the device set of SBKP-1.1 (SBKP-ECG-PPG paired system prototype 1.1. version) after integration (ii), and its representative photo of SBKP-1.1, monitoring human vital signals wirelessly (iii). **b** Representative pulse waves obtained from PPG and SBKP simultaneously. The pulse waves of the SBKP were collected from three different positions of the human body such as **c** wrist, **d** cheek, and **e** neck. Also, PPG and ECG signals were collected simultaneously for verification. **f** The measured heart rate every hour during the daily routine of subject #7 using SBKP-1.1.

The fully integrated SBKP device has been developed, as shown in Fig. 3. We applied our SBKP sensing mechanism to the pulse-wave detection generated from the blood flow of a human heartbeat. A 40-year-old male participant (subject #7) was recruited for the demonstration. As shown in Fig. 3a(i), when the human heart contracts and expands (i.e., systole and diastole), blood flow is generated with a packet-delivering feature. These blood packets generate a very decent mechanical pulse (or beat) and push the SBKP, causing the PET electron donor layer to move closer to the PTFE, producing voltage outputs. The developed compact SBKP device, shown in Fig. 3a(ii), is integrated with ECG, and PPG sensors. A sampling rate of 400 Hz was utilized. Additionally, a grounded electrode was employed, which was attached to any position of the skin on human participants to remove unknown noises such as capacitive or electromagnetic interferences including the surface charge of the skin^50^. For further information regarding the electronic setup to develop the SBKP device, please see Supplementary Fig 4, 5. We termed this device as SBKP prototype version 1.1 (SBKP-1.1). The SBKP-1.1 was tested with human participants as shown in Fig. 3a(iii). Representative video files, such as Supplementary Movies 3, 4(i) for the naked circuit and the compact devices, respectively, are included. As shown in Supplementary Movie 4, the SBKP-1.1 supports a Bluetooth system, allowing for easy measurement of pulse waves at different positions of the human body wirelessly. The detected pulse wave signals are represented in Fig. 3b after inverting the raw signals. It is well-known that signals obtained by mechanical pulses are very similar to the optoelectronic signals of conventional PPG sensors^58^. Therefore, our SBKP sensor signals were simultaneously monitored with PPG signals as a reference. Additionally, from the SBKP and PPG signals, we can calculate heart rate values. Using these values continuously, both signals were verified against the heart rate (HR) obtained from ECG signals. It can be concluded that our SBKP-1.1 detected proper pulse wave signals, including P- and D- waves^52^, which show appropriate pairs with reference PPG signals. However, there is a time difference in pulse waves between PPG and SBKP-1.1 due to the signals being detected from different positions on the human body. The signal-detecting performance of the SBKP-1.1 was tested at three different positions on the human body. Figure 3c–e and their corresponding Supplementary Movies 4(ii–iv), show its pulse wave detection on the wrist, cheek, and neck, with verification using ECG and PPG obtained from electrode positions of the wrist and fingertip, respectively. All detected pulse waves were consistently matched with PPG signals, and their signals were verified with ECG signals in real-time. Also, for longer monitoring periods, Fig. 3f shows the measured HR every hour during the daily routine of subject #7 after the lunch break. Hence, it can be expected that our SBKP-1.1 measures appropriate pulse waves at different human body positions near the superficial arteries.

In this study, the amplitude of the pulse wave at different positions is related to many factors, such as the supportive bony structure or the thick tissue, etc^26^. It is noted that the output voltages of the SBKP-1.1 vary when worn against different parts of the human body. We also verified the signal waves using other references PPG and ECG signals simultaneously. By verifying the SBKP using both ECG and PPG sensors, we calculated the *K* values of the SBKP-1.1 signal. The *K* value indicates a characteristic quantity to reflect changes in cardiovascular physiology and pathology, providing clues about the degree of vascular sclerosis as an important physiological indicator in cardiovascular clinical examinations^59^. This value can be calculated using Eq. (3) below^59^.

3 $$K=\frac{{P}_{m}-{P}_{d}}{{P}_{s}-{P}_{d}}$$

4 $${P}_{m}=\frac{1}{T}{\int }_{\!\!\!0}^{T}p\left(t\right){dt}$$

P_m_ is the mean of the pulse wave P(t) curve caused by arterial pressure, which can be obtained from Eq. (4), and T is the duration of the cardiac cycle. P_s_ and P_d_ are the systolic peaks and the diastolic valley points, respectively. The value of K is influenced only by the area of the pulse wave graph, which is not related to the absolute values of P_s_ and P_d_ and is a scalar quantity. The demanded parameters are defined from pulse waves shown in Supplementary Fig. 6. As a result, 0.363, 0.333, and 0.317 of the K values were obtained from the wrist, cheek, and neck, respectively. Moreover, using the K values, the cardiovascular physiological parameters such as artery compliance (AC) and total peripheral resistance (TPR) have been calculated using Eqs. (5) and (6)^59^:

5 $${AC}=\frac{{SV}}{{P}_{s}-{P}_{d}}$$

6 $${TPR}=\frac{{P}_{m}}{{SV}}\times T$$

where SV indicates the stroke volume (in ml) defined by $SV=\frac{0.28}{{K}^{2}}{(P}_{s}-{P}_{d})T$. Also, P_S_, P_d_, HR, and K, were already measured noninvasively and continuously from the pulse wave signal of SBKP-1.1. Merely calculating the K value is not able to represent the whole cardiovascular system because, depending on the peripheral resistance, vascular elasticity, and blood viscosity, the results of Eqs. (3–6) can vary. Also, based on the previously reported research in the area of physiology, it was found that the different physiological conditions can sometimes have the same K value, while AC and TPR were varied. Hence, we demonstrate calculating all three values simultaneously using SKBP-1.1 in Table 1. Furthermore, our wireless monitoring system with SBKP-1.1 allows the measured data to be exported directly to a CSV file, which would help diagnose the health condition of the cardiovascular system or assist in corresponding physiological studies. Before conducting the final goals toward developing a cuffless BP sensor with SBKP-1.1, the automatic system to calculate the pulse transition time (PTT) should be considered. Otherwise, our ultimate goal of continuously monitoring BP in real-time will be far from achievable.

**Table 1:** Results of the cardiovascular health parameters for a 40-year-old male, a hypertensive patient

	K	AC	TPR
Wrist	0.363	1.13	0.50
Cheek	0.333	1.52	0.51
Neck	0.323	1.70	0.37

In general, the time intervals obtained from the ECG-SBKP or PPG are the pulse arrival time (PAT), which is the sum of the PTT and the pre-ejection period (PEP)^15,16^. Therefore, an additional signal process using the S-peak among PQRST of the ECG signals to remove the PEP was employed. As the core algorithm, we used the peak detection library derived from the Pan–Tompkins theorems^60^. SBKP-1.1, integrated PPG and ECG sensors, generates the downward peaks such as their P-wave and S-peak. Also, for proper peak detection, the lag, threshold, and influence of the algorithm have been carefully considered as shown in Supplementary Fig. 7. The raw signals of the three sensors were converted to show only −1, 0, and 1 to represent downward, null, and upward peaks, respectively. For example, the SBKP-1.1 signals are converted to peak-defined signals in Supplementary Fig. 8. Successively, the two different times for downward peak generation were considered as the times for the first and second pulses detected, and its loop was continuously repeated, which is very similar to common HR calculating algorithms. However, in our case, it is necessary to obtain the first time pulse (FTP) detected from all three sensors at once. Therefore, we applied a peak detection algorithm and converted all sensor signals to peak-defined signals (Supplementary Fig. 8). After collecting each first time from all different sensors, the PTT has been defined as absolute values of the time difference between the two FTPs. The PPG and SBKP-1.1 sensors are comparatively easier to apply the peak detection algorithm than the ECG case. ECG signals generate multiple peaks such as PQRST peaks in Supplementary Fig. 9. Therefore, an additional filtering process to obtain the peak-defined signal should be employed. Supplementary Fig. 10 shows the flow chart of the filtering process. Before applying the process, the randomly detected peaks were unable to define the interval time for signal generation as shown in Supplementary Fig. 10(i). After roughening the original ECG signal using an over-filtering process with a 2nd step low-pass filter (cut-off frequencies: 4, and 1 Hz for 1st and 2nd steps, respectively), the S peak foot signal can be detected consistently, as represented in Supplementary Fig. 10(ii) and Supplementary Movie 5. Also, In Supplementary Movies 6(i), (ii), the automatic calculations of PTT for the ECG-SBKP pair before and after applying the additional filtering process have been demonstrated.

### Neckband type earphone for continous cardiovascular health monitoring

**Fig. 4:**
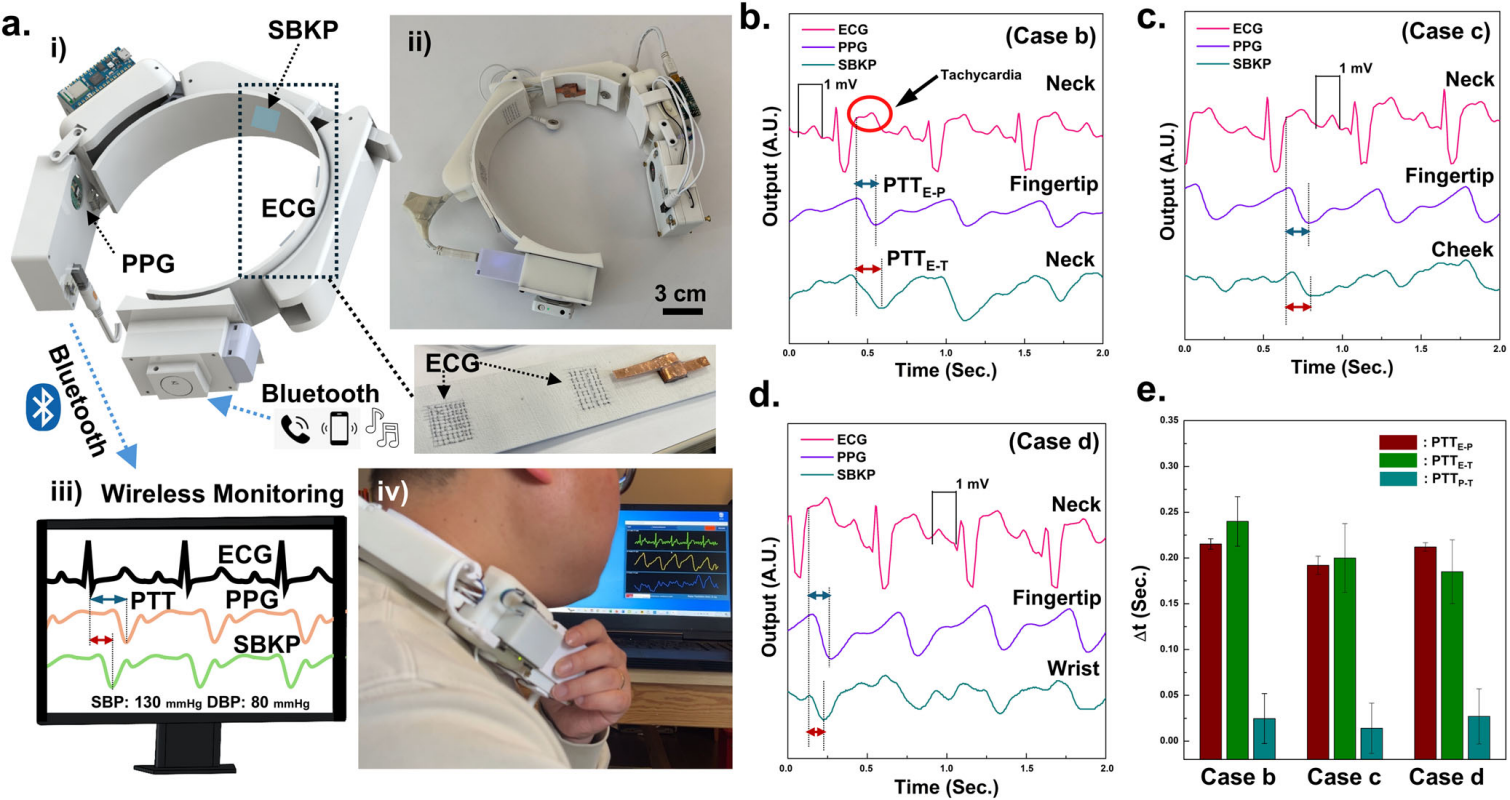
Neckband type earphone for continous cardiovascular health monitoring. **a** Schematics (i, iii) of developed SBKP-1.5 as a wearable neckband-type earphone for continuous diagnosis of cardiovascular symptoms and blood pressure through wireless communication and its photos before (ii) and after (iv) wearing. Three different physiological graphs from three different body positions such as **b** neck, **c** cheek, and **d** wrist, measured by SBKP-1.5. From the graphs, **e** PTT_E-P_, PTT_E-T_, and PTT_P-T_ have been calculated.

The prototype wearable neckband-type earphone for continuous diagnosis of cardiovascular conditions has been developed after fully integrating it with the SBKP-1.1 sensor, as shown in Fig. 4a, and we termed it SBKP-1.5. The ECG and SBKP-1.1 sensing units were integrated into the neckband, and an additional Bluetooth module for smartphone pairing was installed on the portable power storage, as illustrated in Fig. 4a(i), and its actual photo (ii). Also, the additional Bluetooth module includes an output port for personal earphone plug-in, as shown in Supplementary Fig. 10a, allowing human participants to use their earphones according to their preferences. All functions for wireless monitoring are still maintained, so all sensor signals are monitorable through a personal computer, as shown in Fig. 4a(iii), and the actual photo in Fig. 4a(iv) shows the devices after being equipped by a human participant. Additional photos of the device equipped on a human participant are in Supplementary Fig. 10b, c. Also, continuous monitoring with the SBKP-1.5 has been demonstrated in Supplementary Movie 7. Figure 4b–d show the representative SBKP-1.5 signals (the bottom curves) obtained from the neck (case A), cheek (case B), and wrist (Case C) of subject #2, respectively. The ECG signals showed tachycardia, causing an additional peak on the T wave because of the subject’s 118 bpm. By measuring the time interval between the ECG S-peak and the P-wave of the PPG and SBKP, the PTTs for ECG-PPG (PTT_E-P_) and -SBKP (PTT_E-T_) pairs were calculated. Additionally, the PTT from the PPG–SBKP pair (PTT_P-T_) was obtained using both P-waves. The PPTs of the three different pairs are summarized in Fig. 4e. PTT_E-T_ calculated from the neck and cheek showed shorter times than that of PTT_E-P_. In the case of PTT_E-T_ from the wrist, it was vice versa. Such results indicate that the blood flow from the heart to the fingertip is delivered faster than that to the neck or cheek^61,62^. In certain cases, peripheral arteries may exhibit faster or slower transit times due to different mechanical properties, such as elasticity or vessel diameter, or vascular structure variability^63^. Also, PTT_P-T_ from all cases A, B, and C are too short to use, near 0.0 s, which may cause highly fluctuating trends between PTT and actual BP. Therefore, PTT_P-T_ has not been used for the next calibration process for BP measurement.

### Neckband type earphone for continous BP monitoring

**Fig. 5:**
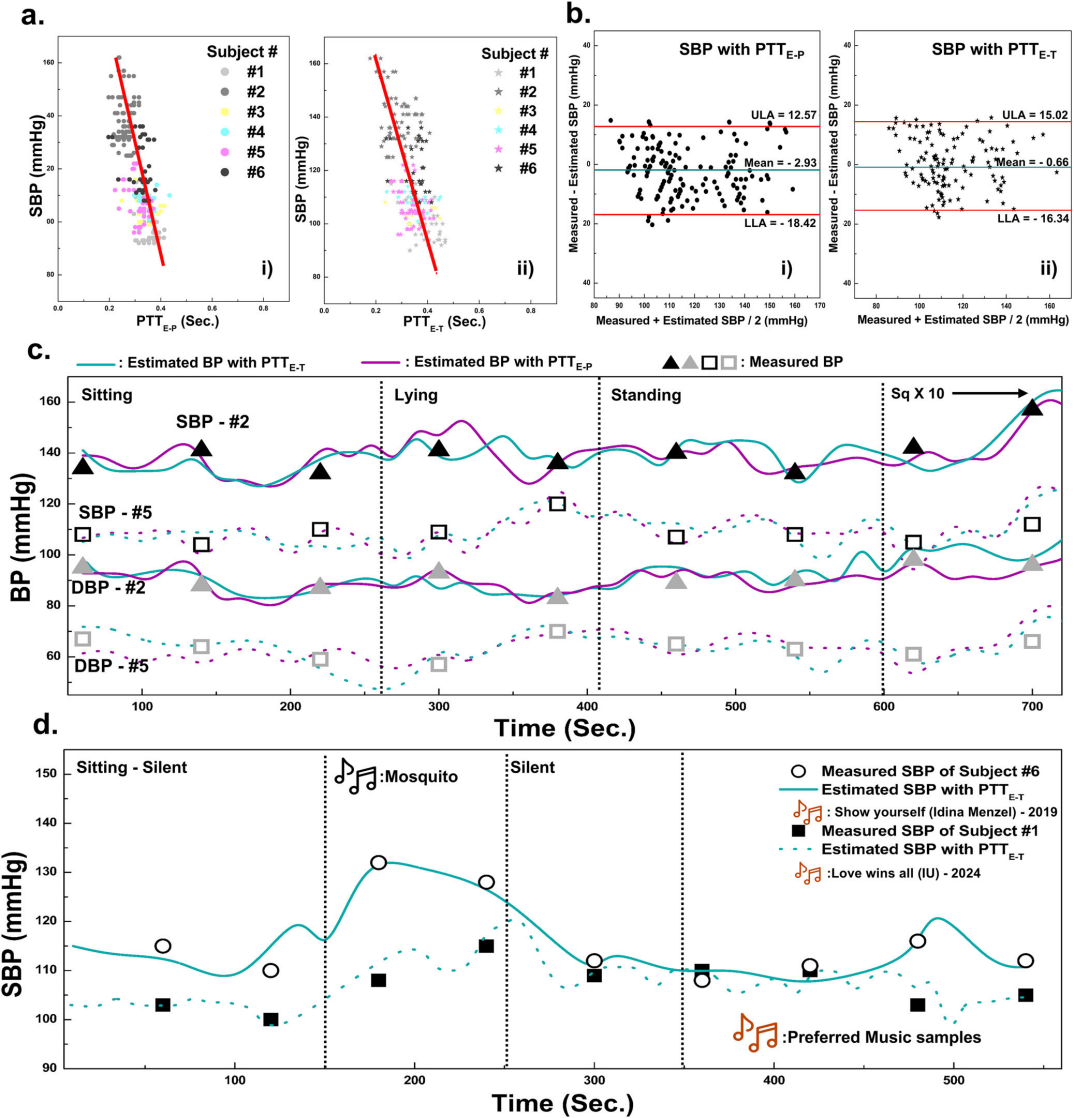
Neckband type earphone for continous BP monitoring. **a** Relationships between PTT_E-P_ (i), PTT_E-T_ (ii), and Systolic BP, and **b** their relevant Bland-Altman plots. Continuous BP monitoring using SBKP-1.5 for subjects #2 and #5 at **c** different human postures and subjects #1 and #6 (**d**) at different music listening conditions. Also, their reference BP has been measured using the sphygmomanometer every 80 and 60 s, respectively.

With six human participants, the trends between systolic BP (SBP), PTT_E-P_, and PTT_E-T_ were obtained, and their corresponding trends for diastolic BP (DBP) are in Supplementary Fig. 11. Regarding the calibration process for the SBKP-1.5, we used the Eq. (7)^16^:

7 $$BP=a{PTT}+b$$

where a and b represent calibration coefficients. The pressure in liquid flow is inversely proportional to the flow rate, which is the Bernoulli principle. Also, BP and PTT have an inversely proportional relationship because they reflect liquid pressure and flow rate, respectively. Moreover, the elasticity of the blood vessels will not change significantly within the period during pulse deliveries or PTT duration. Therefore, it has been proved that such elasticity would be considered negligible when determining the correlation between BP and PTT^18,20^, and the logarithmic model has been applied for the calibration process. To obtain the coefficients a and b in Eq. (7), BP and PTT were collected from the six subjects using a cuff-type sphygmomanometer and SBKP-1.5. Also, we used the PTT values at the same time as BP measured from the cuff-type sphygmomanometer (‘measured BP’). Then, around 250 plots of BP versus PTT_E-P_, and PTT_E-T_ were obtained, as shown in Fig. 5a(i), (ii). From the scatter plots, we estimated a and b using linear square fit, enabling BP estimation when only PTT is measured, termed as ‘estimated BP.’ To analyze the validity of the estimated and measured BPs, the Bland–Altman plot^16,18^ was employed in Fig. 5b(i), (ii) for estimated BP using PTT_E-P_ and PTT_E-T_, respectively. Their corresponding mean differences and standard deviations to estimate the SBP and DBP are summarized in Table 2. They are ≤5 mmHg and ≤8 mmHg for mean differences and standard deviation, respectively, satisfying the criteria of the Association for the Advancement of Medical Instrumentation (AAMI)^11,16^. Therefore, we concluded that the estimated BPs were valid for additional BP monitoring of the subject under different postures and acoustic conditions. Moreover, we collected the PTT variation and variability^62^ of the human subjects in Table 3. Depending on the PTT variation, human cardiac health conditions can be inferred. Based on survey results summarized in Table 4, we can see that higher PTT variations are observed from the subjects who reported their age as above 45 years old. However, the above validation process may not guarantee accurate BP estimation for all individuals, as human bodies exhibit diverse blood vessel configurations due to factors such as congenital conditions, chronic cardiac issues, and professional athletic experience. Consequently, developing BP estimation algorithms or models tailored to these variations remains an important research focus in medical or physiological areas^64^. However, such considerations are beyond the scope of this study.

**Table 2:** Summarized results of the mean and standard deviation of differences after the calibration process to estimate the BP using PTT_E-P_ and PTT_E-T_

Estimated Methods with	PTT_E-P_	PTT_E-T_
**1. Mean of the Difference for**		
Sys BP	−2.93	−0.66
Dia BP	4.97	−4.45
**2. Standard Deviation of the Difference for**		
Sys BP	7.91	8.00
Dia BP	7.72	7.76

**Table 3:** The collected PTT variation and variability correlated to the BP of the human subjects using PTT_E-P_ and PTT_E-T_

	PTT_E-P_		PTT_E-T_	
Subject #	PTT variation (ms/mmHg) for		PTT variation (ms/mmHg) for	
	SBP	DBP	SBP	DBP
1	3.84 ± 1.86	3.09 ± 1.37	3.07 ± 0.81	3.78 ± 0.93
2	2.51 ± 0.54	3.11 ± 0.26	2.99 ± 0.30	3.77 ± 0.35
3	4.45 ± 3.47	4.26 ± 0.66	4.33 ± 0.62	5.45 ± 0.95
4	3.63 ± 1.93	4.90 ± 0.86	3.43 ± 0.64	4.21 ± 0.78
5	3.69 ± 0.85	3.22 ± 0.41	2.75 ± 0.61	4.20 ± 0.48
6	4.29 ± 3.54	2.38 ± 0.86	3.65 ± 0.94	3.71 ± 0.58
Subject #	PTT variability (ms) for		PTT variability (ms) for	
	SBP	DBP	SBP	DBP
1	29.20 ± 15.4	33.50 ± 19.1	57.25 ± 6.72	61.33 ± 15.01
2	70.73 ± 7.75	47.73 ± 5.88	75.65 ± 18.9	57.67 ± 3.21
3	51.30 ± 4.24	35.93 ± 9.53	70.67 ± 8.33	48.90 ± 8.63
4	39.70 ± 19.5	42.23 ± 4.31	46.45 ± 10.9	67.47 ± 10.8
5	63.50 ± 8.54	49.91 ± 13.6	56.21 ± 13.3	86.25 ± 12.8
6	55.40 ± 6.24	45.93 ± 14.9	87.73 ± 22.6	68.02 ± 4.45

**Table 4:** Subject information for the studies with SBKP-1.5 and -1.1

Subject #	Sex	Age	Athletes experience	Current cardiac-relevant problem	Second test type*
**SBKP-1.5**					
1	Female	37	No	No	2
2	Male	39	No	Takotsubo cardiomyopathy	1
3	Male	50	No	No	1
4	Female	47	No	No	1
5	Male	19	Ice hockey	No	1
6	Female	45	No	Hypertension*	2
**SBKP-1.1**					
7	Male	40	No	Hypertension	N/A

*Second test type: BP monitoring at (1) different postures and (2) music listening conditions.

*Hypertension: Subject #6 has been taking blood pressure medication regularly.

It is highly expected that the cuffless type BP monitoring using wearable SBKP-1.5 will allow continuous BP tracking, as it only requires simple contact with the human body. In contrast, conventional cuff-type sphygmomanometers require cuff-contraction/expansion, resulting in void BP measurement times. Our continuous BP monitoring will be capable of recording more detailed BP trends in different postures, even including void time cases of the conventional cuff-type sphygmomanometers. Such features would be advantageous for preventing possible accidents caused by BP-derived problems depending on the postures such as vasovagal syncope and orthostatic hypotension for in-home care patients^65^. Hence, we demonstrated continuous BP monitoring using SBKP-1.5 with referencing BP from the conventional cuff-type sphygmomanometer in the different postures of the human participants as the representative applications. Figure 5c represents the continuous BP monitoring condition of human subjects #2 and #5 with SBPK-1.5, while the sphygmomanometer was used simultaneously as reference BP. The SBKP-1.5 measured the BP of subjects in a real-time, while the conventional sphygmomanometer was available only every 80 s. The results showed that both estimated BP using PTT_E-P_ and PTT_E-T_ are in good agreement with measured BP. Although the estimated BP with PTT_E-P_ and PTT_E-T_ did not fully match each other, their trends for BP variation were similar. For example, subject #2 had 139 and 141 mmHg of SBP for the estimated BP with PTT_E-P_ and PTT_E-T_, respectively, in the initial sitting posture of subject #2, but after 130 s, their differences increased to 4 mmHg. After 325 and 350 s in the lying posture, the differences reached 11 and 14 mmHg. Such differences imply that BP variation depending on different postures would lead to partial BP changes sequentially, not all at once. Also, a pillow during lying posture may keep the head slightly elevated, contributing to less BP fluctuation. Note that PTT_E-T_ measures the time between the electric trigger signals of the heart and the actual pulse delivered time to the neck^15^. In contrast, the blood flow path between the heart and fingertip may face abrupt changes from vertical to parallel flow after lying down, reflecting that estimated BP with PTT_E-P_ may fluctuate more. Such results support the importance of using the pillow for sleeping^66^ or the effects of hand-standing exercise^67^. After returning to a standing posture, the BP of subjects #2 and #5 reverted to about 136 mmHg, and the BP fluctuation difference between estimated BP with PTT_E-P_ and PTT_E-T_, moderated to about 1.2 mmHg. After starting the squat-down exercise 10 times, both subjects showed upward trends in their BP.

Beyond continuous BP monitoring, a further advanced function has been pursued with our SBKP-1.5, designed as a wearable neckband-type earphone, termed an ‘earable’ device. This feature includes potential user convenience add-ons such as phone calls and music listening. Among the possible demonstrations with SBKP-1.5, the effectiveness of music on BP has been explored in this paper as a possible auxiliary cardiovascular therapy system beyond simple BP monitoring. Music, with its complex combinations, generates various stimuli in the human brain, causing physiological changes^68^. Therefore, we expected fluctuations in BP trends depending on the music types, and the advantage of continuous BP monitoring remains effective in collecting detailed BP variations compared to the conventional sphygmomanometer.

Our SBKP-1.5 has an additional function for pairing with a smartphone through the extra Bluetooth module, which allows us to demonstrate further BP monitoring of human subjects at different music listening conditions, as shown in Fig. 5d. Before starting the test, we surveyed participants’ favorite music. Additionally, as a representative sound to affect stress levels, repeating mosquito sound for 100 s was employed. To minimize any external interference affecting their BP, we used the estimated BP with PTT_E-T_ and measured BP using a sphygmomanometer with 60 s measurement intervals as the reference. We also considered the opinions and comments of the subjects during the test. When the subjects were exposed to mosquito sounds, upward trends in their BP were observed. However, subject #6 shows a dramatic increase in the BP from 113 to 132 mmHg compared to subject #1, whose BP increased from 100 to 107 mmHg. According to subject #6’s comments, our pre-notice of mosquito sound listening might have affected her stress level in advance, whereas subject #1 commented that she did not care too much about the upcoming sounds. After 100 s of silence, their BP returned to 108 and 110 mmHg for subjects #1 and #6, respectively. When the subjects listened to their favorite music, subjects #1 and #6 showed different BP variation patterns. Subject #6 maintained a stable BP trend initially, but after around 140 s, their BP reached 121 mmHg. Although subject #1 showed fluctuating BP trends during their favorite music listening, after 150 s, their BP dropped to 98 mmHg. The changes in their BP during favorite music play, particularly during highlight parts or main peaks before the finale, could be the main reason for such variations. However, clarifying different BP trends during favorite music listening remains challenging, as these variations may be influenced by individual characteristics or the psychological effects of specific music genres or themes^68,69^. Nevertheless, our tests provide clues about the effectiveness of music not only on emotions but also on cardiovascular health^69^. Additionally, there is a wide range of studies that investigated the effect of music listening on enhancing blood flow^69,70^. Conducting such studies in depth is beyond our scope, but we demonstrated the relationship between BP and music listening as a sample application to suggest the potential benefits of the continuous BP monitoring system through our proposed SBKP-1.5 for further extended studies in cardiovascular health-relevant areas.

## Discussion

We developed a wearable neckband-type earphone for the continuous monitoring of cardiovascular symptoms and BP. Inspired by knotting crafts, the self-powered box knot pulse wave sensor (SBKP) that utilizes a triboelectric sensing mechanism was developed. The knotting methods allowed us to generate a multi-stacked structure, which contributed to increasing the total effective area between interlaced strips while the top view area formed by the first knotting layer was still small. This architectured SBKP showed a 26% higher voltage output than the plane-type sensing unit at the same apparent area for forming triboelectric pairs. Additionally, its self-powering performance was evaluated using two different criteria: Voltage output depending on (i) load resistance and (ii) capacitance. Furthermore, the generated power and energy were calculated. Beyond the triboelectric performances, the knotting architecture of SBKP exhibited excellent sensitivity, featuring 38.17 mV Pa^−1^ with a 0.006 kPa resolution, indicating a promising capability to measure low mechanical forces, such as human pulse waves. Moreover, our research was conducted from a material-independent perspective. Thus, after incorporating other in-depth material approaches, superior performance of the SBKP is expected. As a first step toward developing a wearable device for the continuous monitoring of cardiovascular symptoms and BP, the SBKP was integrated with ECG and PPG sensors, and a compact human vital signal sensing device was fabricated, termed SBKP-1.1. SBKP-1.1 measured three different sensor signals simultaneously, and its wireless connection allowed continuous real-time monitoring of cardiovascular symptoms. This SBKP-1.1 was further upgraded to create a wearable neckband-type earphone for continuous diagnosis of cardiovascular symptoms and BP, named SBKP-1.5. Additional functions such as a Bluetooth pairing system with a smartphone for music listening or phone calls, and an automatic PTT calculation algorithm were included in SBKP-1.5. To add the BP measurement function to SBKP-1.5, a calibration process was conducted with six human participants. The data, including PTTs of ECG-PPG and ECG-SBKP pairs with their corresponding BP measured using a conventional sphygmomanometer, were collected in real-time. Based on the trends between PTTs and BP, the firmware of SBKP-1.5 was updated to measure BP directly. After the calibration process, we demonstrated the advantages of a wearable neckband-type earphone for continuous BP monitoring through SKBP-1.5. Our SKBP-1.5 can measure the BP without delays, whereas a sphygmomanometer requires at least 60 s for each BP measurement. These features allowed more careful BP monitoring of human subjects in different postures and acoustic conditions. The developed wearable neckband-type earphone for human healthcare assistance, which we refer to as an “earable healthcare device,” would be beneficial for personal healthcare, even during remote working conditions. Beyond out-of-clinic care, such earable devices may provide further meaningful studies in the area of music therapies for cardiovascular health, such as in-situ variations in human BP under different music listening conditions.

## Methods

### Research ethical approvals

All procedures to perform the studies of the wearable neckband-type earphone for human healthcare assistance with SBKP-1.1 and −1.5 have been approved by the research ethics committee at Simon Fraser University in Canada: REB#: 30002212 – Minimal Risk. All participants provided informed consent before participating in the experiments for the calibration process of our SBKP-1.5 and the publication of data. The signed consent for photography and movies during the test was obtained from the individual. According to the legal term of Canada, the confidentiality of participants is strictly respected.

### Fabrication of SBKP

PET and PTFE strips were collected from a plastic file folder and Teflon tape, respectively. After cutting the PET file folder with the size of 7 × 200 mm^2^ to make the PET strip, commercial copper tape was attached to the surface, and then the other PET strip was covered, which is used as one triboelectric layer. Also, the other triboelectric pair has been prepared with the copper tape warped by PTFE tape. Both strips were cleaned using subsequently by menthol, isopropyl alcohol, and deionized water. All stripes have around ~3 mm thickness. As the next step, the strips were knotted based on the method to make a box knot craft. After fabricating the three-layered structure of the box knot with PTFE and PET strips, the structure was coated using hydrophobic Ecoflex 00-50 that improved to generate triboelectric charges between two strips^71^.

### Experimental set-up to evaluate the performance of SBKP

A customized pressure gauge was prepared using a force sensor of a commercially available digital scale (Springfield Instruments Inc.) with 0.01 g units of measurement. The SBKP sensing unit was positioned on the tray of the customized pressure gauge, and integrated into the oscilloscope to measure the electrical output of the unit (DSO3102A, Agilent Technologies, Inc.). When the external pressure was applied, the g/cm^2^ was obtained and converted to kPa. The vibration (or periodic impact) was applied using the customized actuator obtained from a motor and gears in a commercial handheld electric muscle massager machine (Olsky, model- M68-7), and the applying force or speed was controlled using a function generator (Siglent, SDG1032X). Moreover, the voltage output of SBKP was evaluated using two different equivalent circuits that are shown in Fig. 2a.

### Preparation of SBKP-1.1

All external frames for housing SBKP-1.1 have been prepared using a 3D printer (Ender3-pro) with commercially available PETG filament. Also, its relevant computer-aided designs (CAD) have been prepared using Solidworks 2023 software and converted to g-code for the 3D printing process. The commercial PPG and ECG sensor (MAX86150, Agilent Technologies, Inc., and integrated by Protocentral Electronics) has been integrated with SBKP. The force generated by the heartbeat is very small, producing SBKP signals in the mV range. Regarding the analog signal processing to minimize the noise of SBKP, using an operational amplifier (LM358P) for low-pass filtering, the analog signal was delivered to an Arduino Nano, where it was digitalized, squared, and filtered using two cut-off frequencies. A 1st-order filter was applied to remove unknown or ambient noise. Then, digital filtering was applied to smooth the graphs after digitally amplifying the signals with a cut-off frequency of 6 Hz. The final filtering at 3 Hz was used, as our sensor targets heart-beat measurements (1 ~ 3 Hz). Also, digital signal processing has been applied using a programmed algorithm (C/C + +) in the Arduino Nano platform. For the continuous monitoring of sensor signals simultaneously, packet-based serial communication has been employed. Also, source code can be obtained from the website of Protocentral Electronics. Moreover, the Bluetooth module (HC-05) was integrated and communicated with Arduino Nano via the hardware serials. The wireless signal from the Bluetooth module was connected to the computer using a virtual USB serial port setup. Based on the example code provided by Protocentral Electronics, the customized displaying system in personal computers using Processing 4 software (Java) has been prepared.

### Preparation of SBKP-1.5

All frames for the SBKP-1.5 were prepared using a 3D printer (Ender 3-pro) as same as the case of SBKP-1.1. The neckband of the SBKP-1.5 was prepared using 3D printing with TPU filament and a commercial band (NellcorTM, Covidien Ltd) has been inserted into the 3D printed neckband for its size adjustability. Also, a commercially available conductive wire (bead landing, Michaels) was weaved on the neckband to fabricate ECG dry electrodes, and integrated into the ECG sensor (Signal-to-noise ratio, SNR: 24.34 ± 0.53 dB) of SBKP-1.1. Also, the SBKP sensing unit and its grounding pair were equipped on the neckband, and connected to SBKP-1.1. For the additional functions such as music listening and phone calls of SBKP-1.5, A Bluetooth module (SBH20, Sony Inc.) was integrated into the SBKP system, which allowed the subjects to use their earphones through the plug-in in the module. A medical-graded sphygmomanometer (Equate^TM^ – 4000 series, Walmart Canada Inc.) was employed for the calibration process of the SBKP-1.5 to add the BP measuring function and used as a standard BP reference for continuous BP monitoring of human subjects using the calibrated SBKP-1.5.

### Human subject studies

The two step-wise human subject studies were conducted. From the first tests with all human subjects, PTT values and reference BP were collected altogether from SBKP-1.5 and cuff-type sphygmomanometer, respectively. Around 250 plots were collected to estimate the trends between PTT values and BP from all subjects. The dry electrodes and SBKP sensing units for ECG and pulse wave sensing, respectively, on the neck bands of the SBKP-1.5 were positioned naturally once the subjects wore the SBKP-1.5. Also, their fingertips were placed on the sensing units of the PPG sensor on the edge of the SBKP-1.5. During the test, The subjects were requested to take three different postures such as sitting, lying, and standing for 200, 150, and 200 s, respectively, after resting for 50 s, perform 10 times of squat for the additional data with faster HR. As the second step of the test, based on the survey results, the subjects joined two different studies: Monitoring BP using SBKP-1.5 at (1) different postures and 10 times squats with the same test procedure conducted for the calibration process, and (2) different music listening conditions. For the BP monitoring test at different music listening, the subjects’ favorite music was surveyed, and their BP was monitored in a series of silent acoustic, mosquito sound, silent acoustic again, and their favorite music listening conditions for 150, 200, 100, and 200 s, respectively. In order to minimize the unwanted acoustic conditions, we used personal in-ear-type headphones.

## Supplementary information

Checklist_npj_bio_Inno_ep

PRISMA_2020_checklist

Supplementary Information_SBKP_Revised_Dec_24

Supplementary Movie 1

Supplementary Movie 2

Supplementary Movie 3

Supplementary Movie 4

Supplementary Movie 5

Supplementary Movie 6

Supplementary Movie 7

## Data Availability

Data including source or pseudo codes and electronic designs is provided within the manuscript or supplementary information. The detailed data that support the findings of this study are available from the corresponding author upon reasonable request.

## Code availability

Firmware to operate the SBKP-1.1 or -1.5 in our studies is programmed using the open source codes from https://github.com/Protocentral/protocentral_max86150_ecg_ppg. Also, the fully customized codes of this study are presented as pseudo codes in the supplementary information, and available from the corresponding author upon reasonable request.
