# Supplementary material for: Neckband-type earphone for continuous monitoring of cardiovascular symptoms via self-powered box knot pulse-wave sensor
Source: NPJ Biomed Innov. 2025 Feb 12;2:4. doi: 10.1038/s44385-025-00007-z (PMC13055091; doi:10.1038/s44385-025-00007-z)
Supplement: Supplementary file 3 — Supplementary Information_SBKP_Revised_Dec_24 [file 44385_2025_7_MOESM3_ESM.pdf]

Supplementary Information

Neckband Type Earphone for Continous Monitoring of Cardiovascular Symptoms via Box Knot Self-powered Pulsewave Sensor

Tae-Ho Kim[1,2], *Dominic Jaworski*[1,2], *Rakesh Sethi*[3], *Elise Huisman*[3], *Kam Fung*[3], *Edward J. Park*[1,2]*

[1]Biomechatronic Systems Laboratory, School of Mechatronic Systems Engineering, Simon Fraser University, Surrey, BC, Canada

[2]WearTech Lab, Simon Fraser University, Surrey, BC, Canada

[3]Research & Technology and Open Innovation, 6135 Gunbarrel Ave, Boulder, CO, USA, Acute care & Monitoring, Medtronic

[*]Corresponding author - E-mail: ed_park@sfu.ca

Contents

Supplementary Figures:

Supplementary Figure 1. Photos to show the knotting methods to generate Korean traditional box knot architecture. After repeating 1-11 steps, a multi-stacked structure has been generated.

Supplementary Figure 2. (a) Voltage output of SBKP under the two different periodic impacts applying. (b) power density and (c) stored energy at different load resistances and capacitances, respectively, of the SBKP and control samples.

Supplementary Figure 3. Circuit diagram of the C-to-V converter for the C-V compensation of the SBKP to generate a retended voltage signal.

Supplementary Figure 4. The electronic setup to develop the SBKP-1.1 device. For the pseudo code, please refer to the supplementary Note.

Supplementary Figure 5. The circuit design of the MAX86150 development kit and its photo. All copyright reserved by Protocental Electronics Ltd.

Supplementary Figure 6. Representative graphs to define the mean (P_m) obtained from Equation (3), the systolic peaks (P_s), and the diastolic valley (P_d) points.

Supplementary Figure 7. (a) Flow chart to convert the sensor signals to peak-defined signals, and (b) its example.

Supplementary Figure 8. Flow chart to calculate the PTT using three different sensor signals

Supplementary Figure 9. Schematics for ECG signals generating PQRST peaks. Their electrical signals, known as the cardiac conduction system, include the sinoatrial (SA) node, atrioventricular (AV) node, and His-Purkinje system. When the SA node initiates the electrical signals results in the atrial contraction to push the blood into the ventricles. Then, an electrically

impulsed AV node facilitates atriums to be empty. Once its contraction is released, the electrical signals are delivered to the electrical highway called His bundles around the Purkinje fibers that divide into Purkinje fibers connected to cells in the walls of the ventricles. Such phenomena cause the electrical stimulation of the ventricles to let the heart pump oxygenated blood into the arteries. The entire phase is reflected on the QRS complex wave of the ECG signals.

Supplementary Figure 10. (a) Additional filtering process to obtain the peak-defined signals from ECG signals generating PQRST peaks. (b) The randomly detected peaks were unable to define the interval time for signal generation (i). After roughening the original ECG signal using an over-filtering process with 2nd step low-pass filter (Cut-off frequencies: 4, and 1 Hz for the 1st and 2nd steps, respectively), the S peak signal can be detected consistently (ii).

Supplementary Figure 11. Additional photos after equipping the device onto a human participant: (a) left and (b) right side view and (c) sensing units of the device.

Supplementary Figure 12. Relationships between (a) $PTT_{E\text{-}P}$ (i), (b) $PTT_{E\text{-}T}$ (i) and Diastolic BP, and their corresponding Bland-Altman plots (ii).

Supplementary Note. Pseudo code of Arduino programming (C++) for SBKP -1.1 and -1.5.

Supplementary Movies:

Supplementary Movie 1. (mp4 format). LED module with W and T arrangements, integrated with SBKP. During tapping the SBKP, the module has been turned on.

Supplementary Movie 2 (mp4 format). Signals of the SBKP detecting blown wind by mouth

Supplementary Movie 3 (mp4 format). The early stage of SBKP to demonstrate the sensor performances before generating its compact device.

Supplementary Movie 4 (mp4 format). The SBKP-1.1 with wireless communication system (i). Signal-detecting performance of the SBKP-1.1 at three different positions of the human body such as wrist (ii), cheek (iii), and neck (iv).

Supplementary Movie 5 (mp4 format). The consistently detected S peak foot signal after the additional signal process.

Supplementary Movie 6 (mp4 format). The automatic calculations of PTT for the ECG-SBKP pair before (i) and after (ii) applying the additional filtering process. Calculated PTT was presented in the bottom right corner.

Supplementary Movie 7 (mp4 format). Continuous monitoring of ECG, PPG, and SBKP signals with the SBKP-1.5, simultaneously.

Supplementary Note. Pseudo code of Arduino programming (C++) for SBKP -1.1 and -1.5.

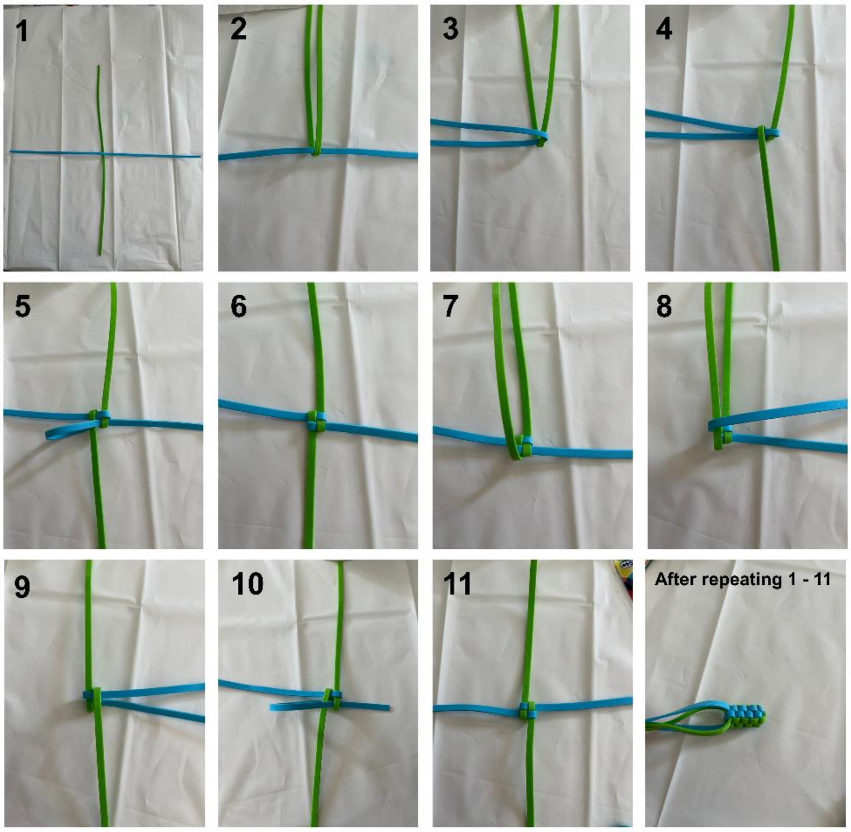

Supplementary Figure 1. Photos to show the knotting methods to generate Korean traditional box knot architecture. After repeating 1-11 steps, a multi-stacked structure has been generated.

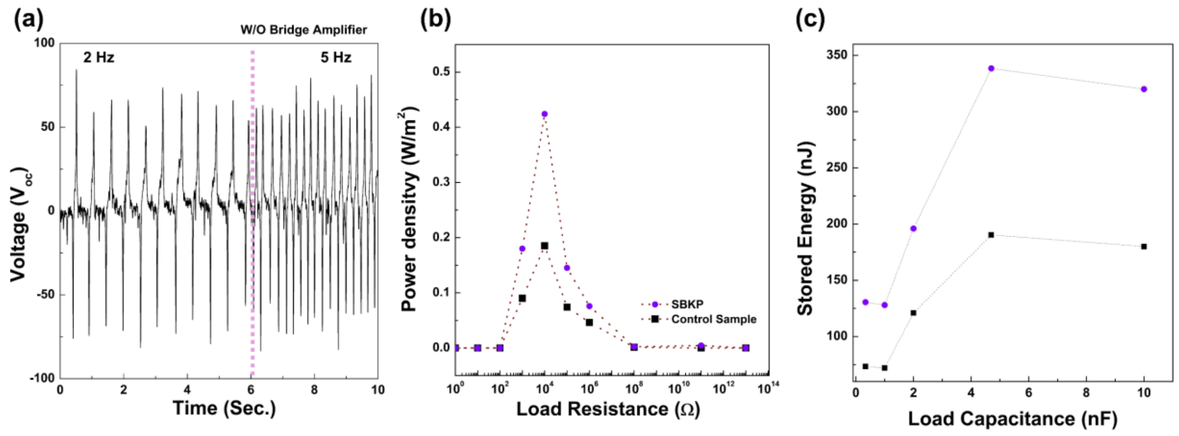

Supplementary Figure 2. (a) Voltage output of the SBKP under two different periodic impacts. (b) Power density and (c) stored energy at different load resistances and capacitances, respectively, of the SBKP and control samples.

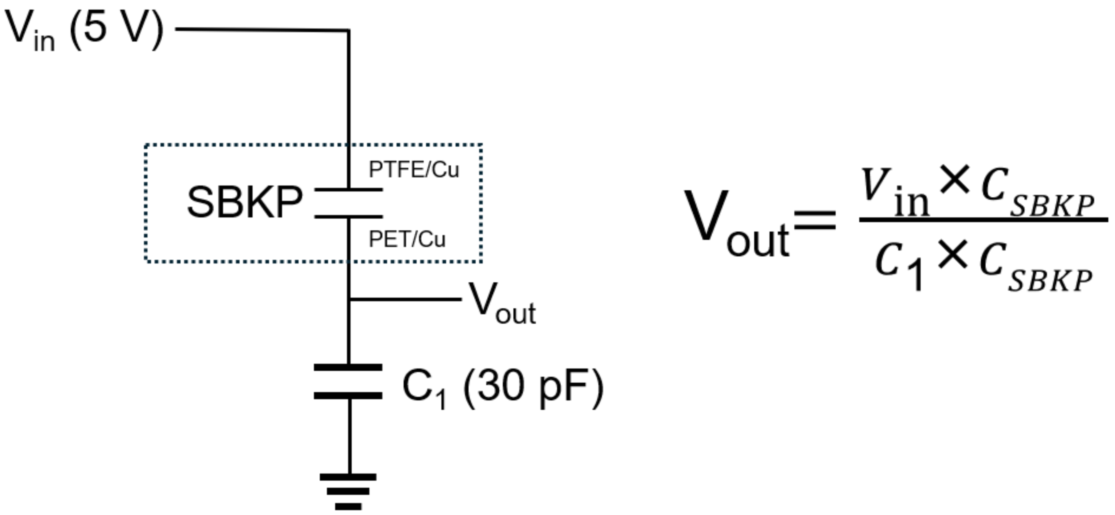

Supplementary Figure 3. Circuit diagram of the C-to-V converter for the C-V compensation of the SBKP to generate a retained voltage signal. Input voltage (V_{in}) and capacitance (C_1) can be adjusted depending on experimental conditions.

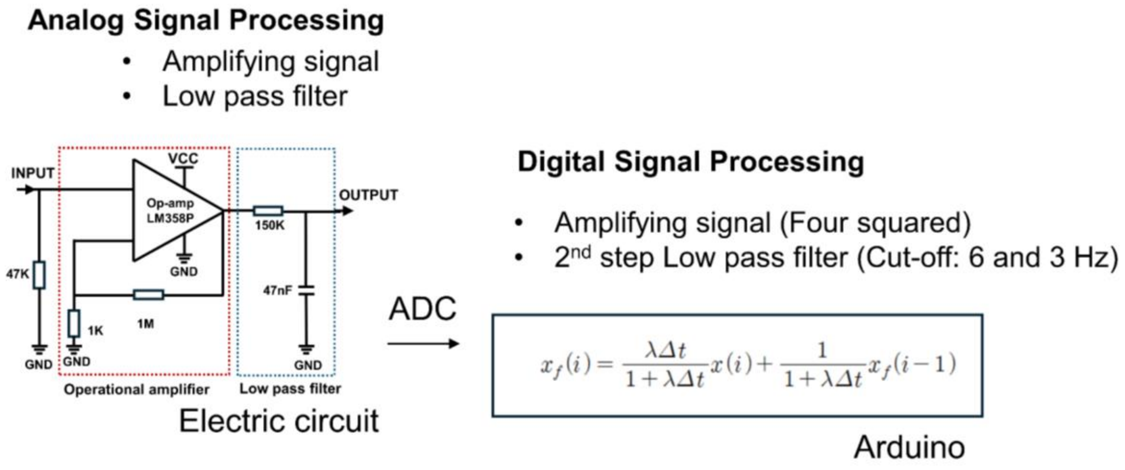

Supplementary Figure 4. The electronic setup to develop the SBKP-1.1 device. For the pseudo code, please refer to the supplementary Note.

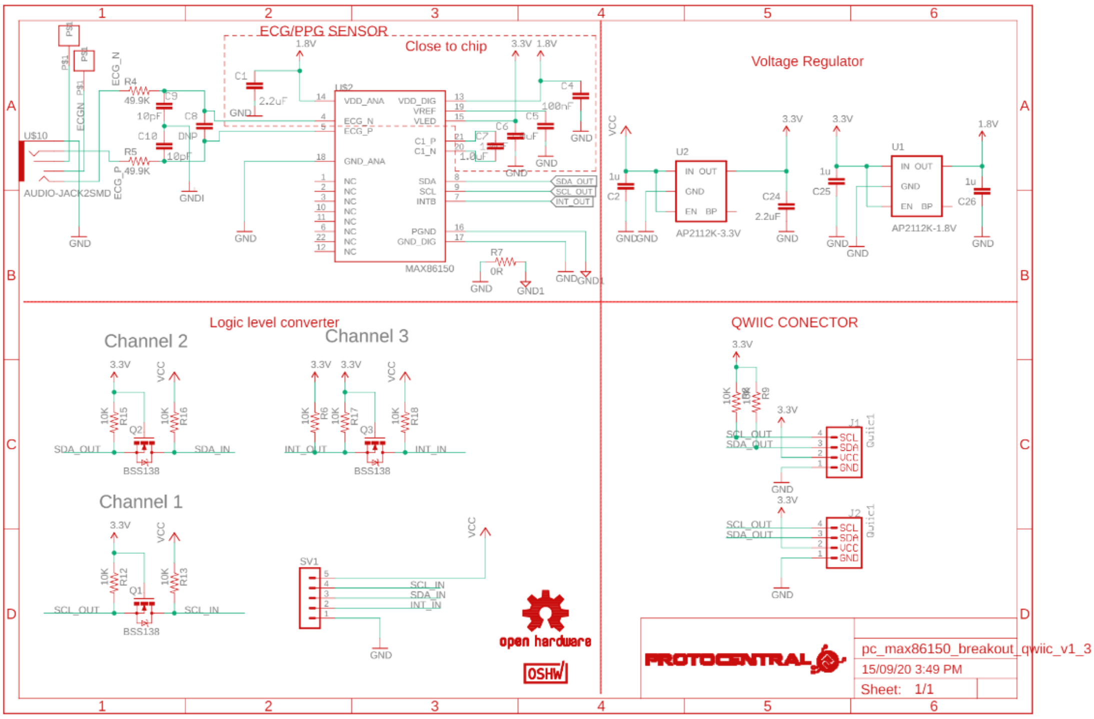

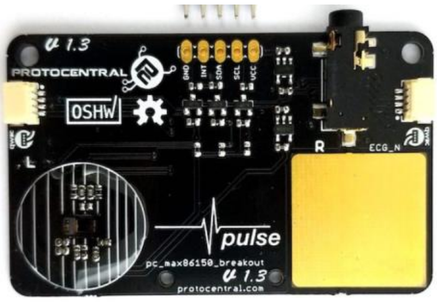

Supplementary Figure 5. Circuit design and photography of the MAX86150 development kit.

All rights reserved by Protocental Electronics Ltd.

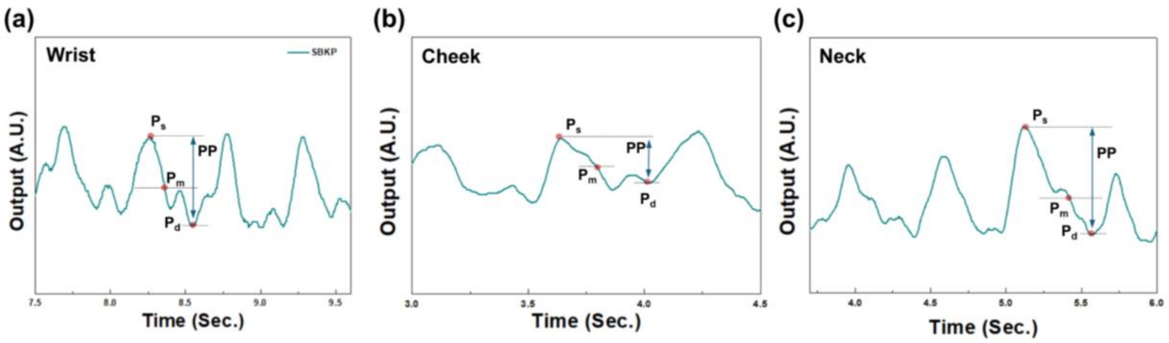

Supplementary Figure 6. Representative graphs to define the mean (P_m), the systolic peaks (P_s), and the diastolic valley (P_d) points for Equation (3) related to (a) wrist, (b) cheek, and (c) neck.

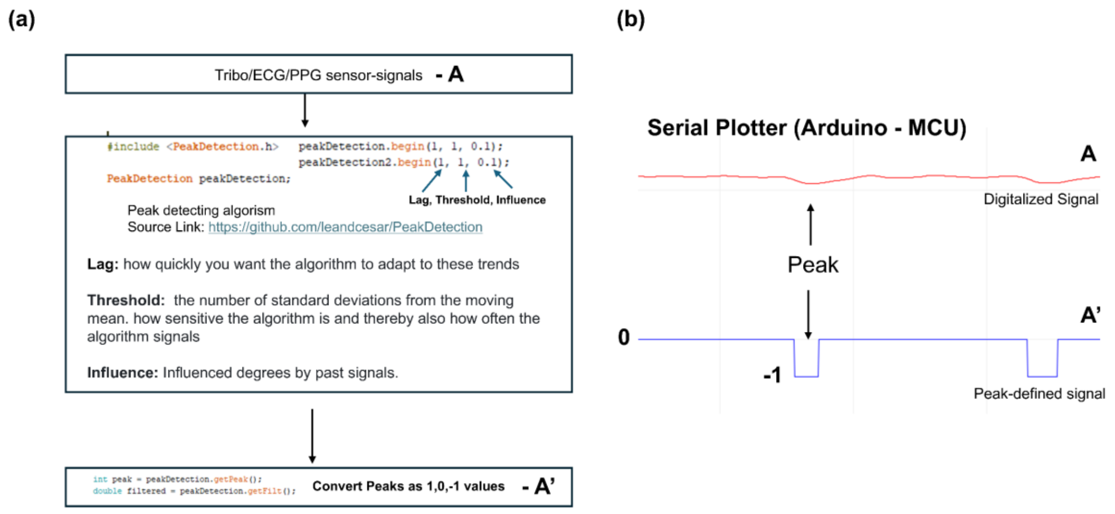

Supplementary Figure 7. (a) Flow chart for converting the sensor signals to peak-defined signals, and (b) an example.

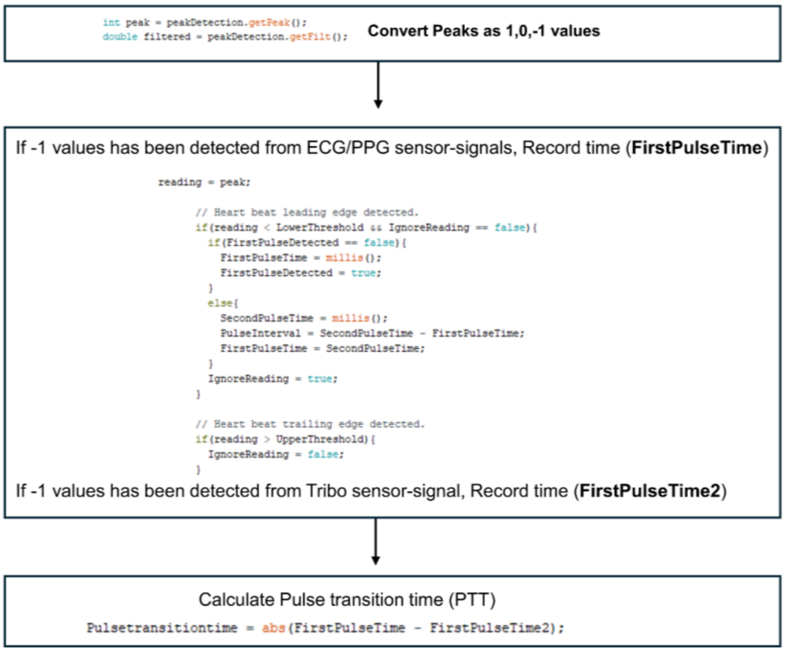

Supplementary Figure 8. Flow chart to calculate the PTT using three different sensor signals.

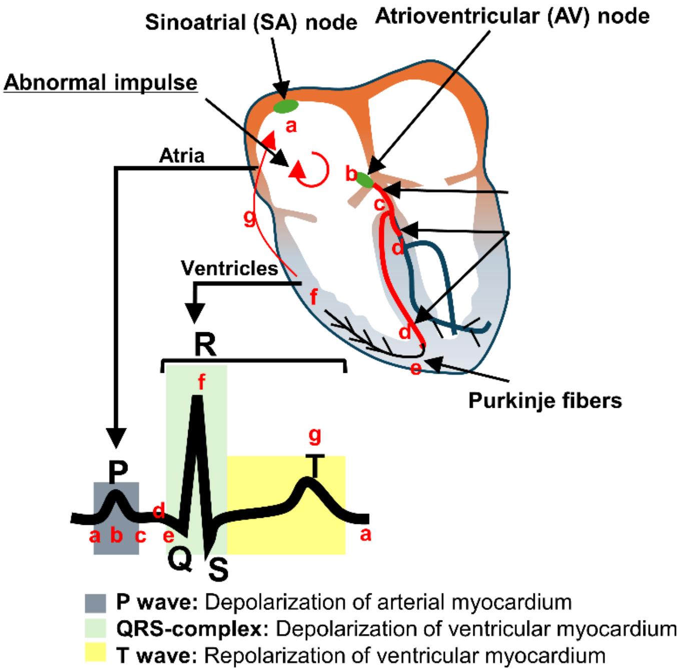

Supplementary Figure 9. Schematics for ECG signals generating PQRST peaks. These electrical signals, part of the cardiac conduction system, involve the sinoatrial (SA) node, atrioventricular (AV) node, and His-Purkinje system. When the SA node initiates electrical signals, it results in atrial contraction to push blood into the ventricles. Then, an electrically impulsed AV node facilitates the emptying of the atria. Once atrial contraction is released, the electrical signals are delivered to the His bundles, which act as the electrical highway and branch into Purkinje fibers connected to cells in the walls of the ventricle. This process stimulates the ventricles to pump oxygenated blood into the arteries. The entire phase is reflected in the QRS complex of the ECG signals.

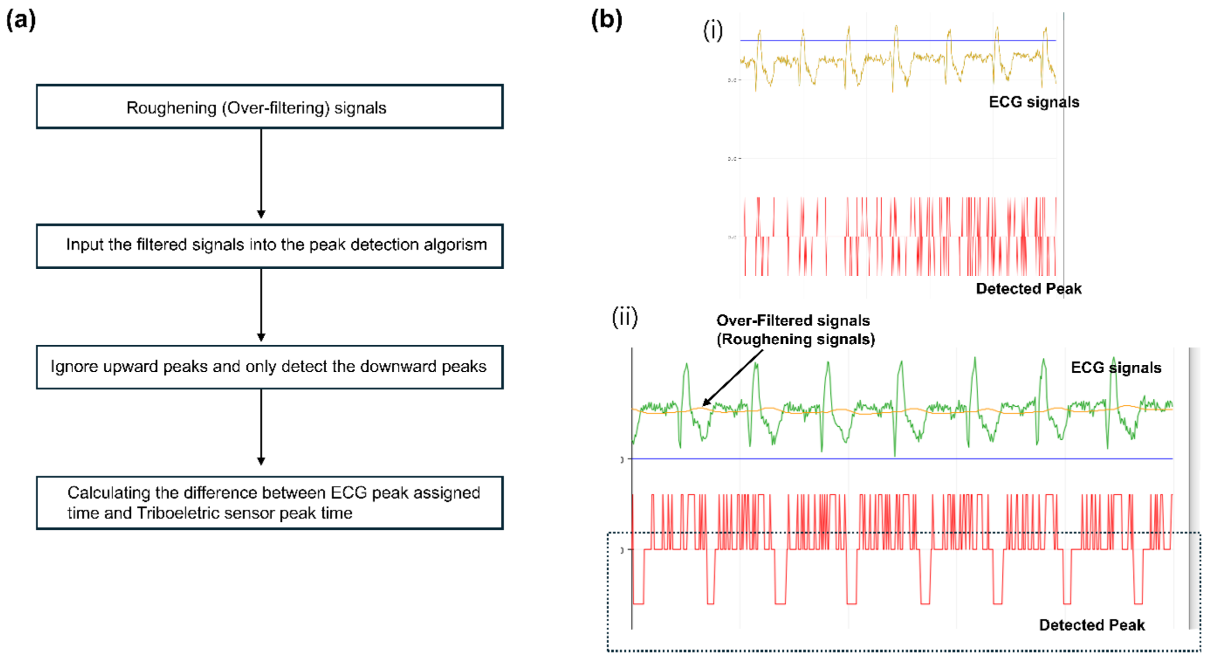

Supplementary Figure 10. (a) Additional filtering process to obtain peak-defined signals from ECG signals generating PQRST peaks. (b) Initially, the randomly detected peaks were unable to define the interval time for signal generation (i). After smoothing the original ECG signal using an over-filtering process with a 2nd-step low-pass filter (cut-off frequencies: 4 and 1 Hz for the 1st and 2nd steps, respectively), the S peak signal can be detected consistently (ii).

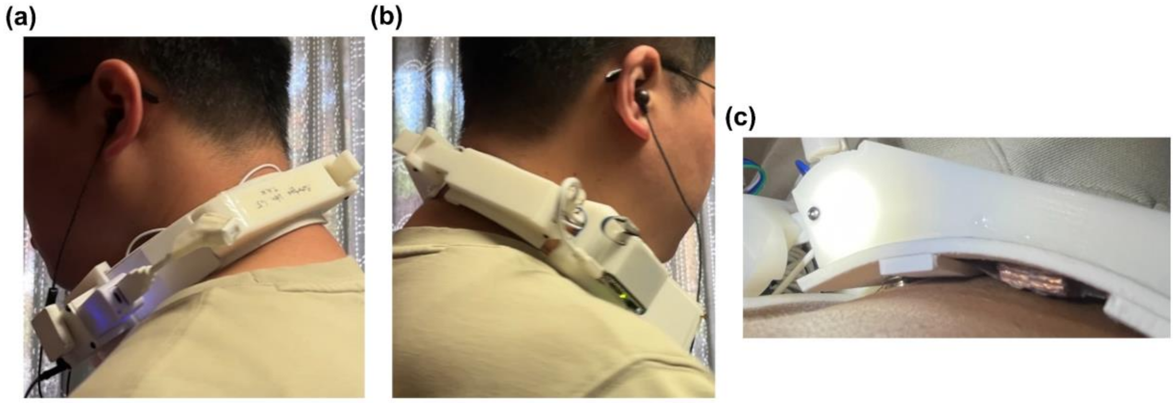

Supplementary Figure 11. Additional photos after equipping the device onto a human participant: (a) left-side view, (b) right-side view, and (c) sensing units of the device.

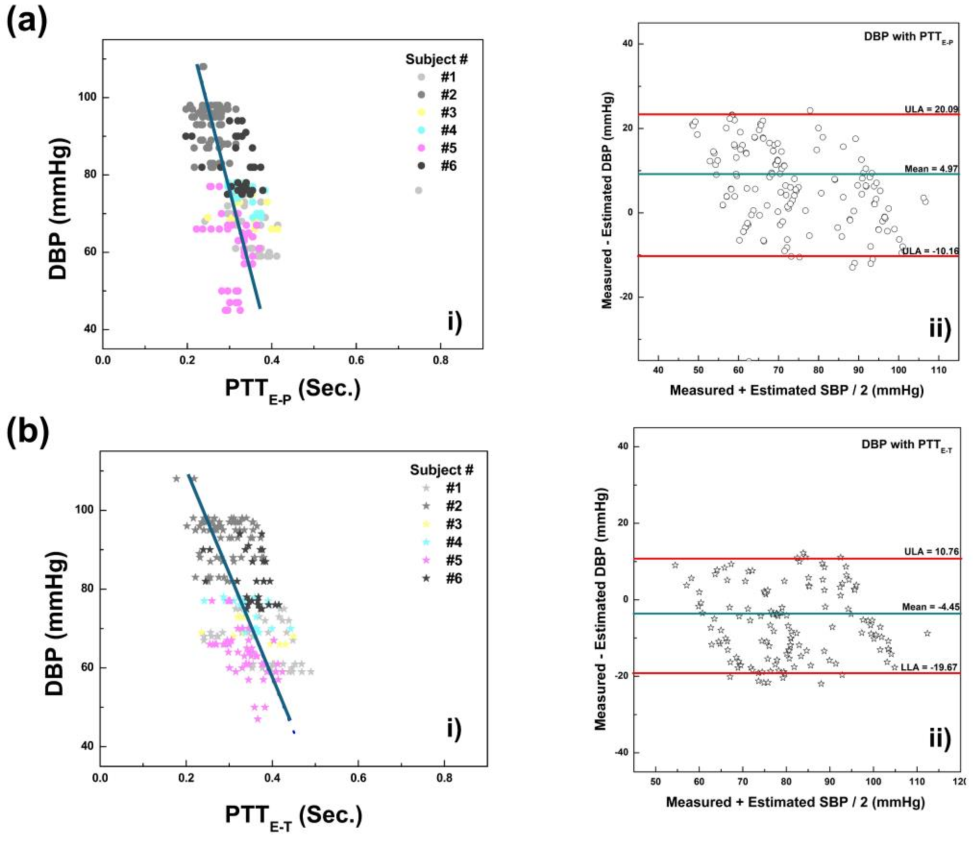

Supplementary Figure 12. Relationships between (a) PTT$_{E-P}$ (i), (b) PTT$_{E-T}$ (i) and Diastolic BP, and their corresponding Bland-Altman plots (ii).

Supplementary Note. Pseudo code of Arduino programming (C++) for SBKP -1.1 and -1.5.

Import necessary libraries

Import Wire
Import MAX86150
Import PeakDetection

Define constants for packet markers, data types, and packet structure:

- Packet start markers: CES_CMDIF_PKT_START_1, CES_CMDIF_PKT_START_2.
- Packet type: CES_CMDIF_TYPE_DATA.
- Packet stop marker: CES_CMDIF_PKT_STOP.
- Data length: DATA_LEN.

Define sampling interval:

- ANALOG_PIN_TIMER_INTERVAL = 2ms.

Define filter parameters:

- Primary filter cutoff frequency (fc = 6 Hz).
- Secondary filter cutoff frequency (fc2 = 3 Hz).
- Sampling time (dt = ANALOG_PIN_TIMER_INTERVAL / 1000).
- Filter lambdas (lambda, lambda2).

Initialize variables:

- Sensor values: sensorvalue, filtervalue, x
- Filter states: x2, x_f, x_f2, x_fold, x_fold2.
- Sensitivity adjustment: sensitivity. // Optional
- Data packet arrays for UART transmission.

// Setup Function

Function setup():

    Initialize serial communication at 57600 baud.
    Print startup message.
    Initialize the MAX86150 sensor:
    If initialization fails:
    Print the error message and stop execution.
    Print the sensor's part ID.
    Configure sensor settings (e.g., LED drive).

// Main Loop

Function loop():
Calculate elapsed time since the last loop iteration.
Update the analog sampling timer.

If the sampling timer interval has passed:

// Sensor Reading from SBKP

Read the analog value from the analog pin (A6):
sensorvalue = analogRead(A6) // optional
x = analogRead(A6)

Update the filtered value using sensitivity adjustment: //optional
filtervalue = filtervalue * (1 - sensitivity) + sensorvalue * sensitivity. : //optional

// Non-linear Transformation // Digital signal processing for SBKP

Compute `x` as a transformed sensor value to be the actual voltage value
Apply the `sq` function 4 times to amplify non-linear effects:

// Low Pass Filtering Process // Digital signal processing for SBKP

Apply first-order filter to `x`:
x_f = (lambda / (1 + lambda)) * x + (1 / (1 + lambda)) * x_fold.
Update the previous state for the primary filter:
x_fold = x_f.

Use the output of the first filter as input for the second filter:
x2 = x_f.
Apply the filter to `x2`:
x_f2 = (lambda2 / (1 + lambda2)) * x2 + (1 / (1 + lambda2)) * x_fold2.
Update the previous state for the secondary filter:
x_fold2 = x_f2.

Map the final filtered value to input_voltage:
input_voltage = x_f2.

// Apply peak detection for each sensor

Add data to peak detection for SBKP sensor
Check if a peak is detected (0, 1, or -1) for SBKP sensor
Retrieve the filtered value from peak detection for SBKP sensor

Add data to peak detection for PPG sensor
Check if a peak is detected (0, 1, or -1) for PPG sensor
Retrieve the filtered value from peak detection for PPG sensor

Add data to peak detection for ECG sensor
Check if a peak is detected (0, 1, or -1) for ECG sensor
Retrieve the filtered value from peak detection for ECG sensor

// Detect heartbeats by checking for peaks above/below the defined thresholds

If a heartbeat is detected for SBKP sensor (using thresholds):
If first pulse detected for SBKP sensor
Record the time of the first pulse (FirstPulseTime)
Else: Record the second pulse time and calculate Pulse Interval for sensor
Reset IgnoreReading flag for SBKP sensor after peak detection

If a heartbeat is detected for PPG sensor (using thresholds):
If first pulse detected for PPG sensor:
Record the time of the first pulse (FirstPulseTime2)
Else: Record the second pulse time and calculate Pulse Interval for PPG sensor
Reset IgnoreReading2 flag for PPG sensor after peak detection

If a heartbeat is detected for ECG sensor (using thresholds):
If first pulse detected for ECG sensor:
Record the time of the first pulse (FirstPulseTime3)
Else: Record the second pulse time and calculate Pulse Interval for ECG sensor
Reset IgnoreReading3 flag for ECG sensor after peak detection

// Calculate BPM for each sensor based on pulse intervals

Set BPM = (1 / PulseInterval) * 60 * 1000   // Calculate BPM for SBKP sensor
Set BPM2 = (1 / PulseInterval2) * 60 * 1000   // Calculate BPM for PPG sensor
Set BPM3 = (1 / PulseInterval3) * 60 * 1000   // Calculate BPM for ECG sensor

// Calculate pulse transition times between different sensors

Set Pulsetransitiontime = Abs(FirstPulseTime3 - FirstPulseTime2)
Set Pulsetransitiontime2 = Abs(FirstPulseTime - FirstPulseTime3)

// Sensor Data Processing

If new data is available from the MAX86150 sensor:
Retrieve sensor values:
- irunsigned16 = IR data (PPG).
- SBKP16 = input_voltage (filtered SBKP signal).
- ecgsigned16 = ECG data.

- intervaltime6 = Pulsetransitiontime2 (calculated pulse transition time)

Send the data through UART:
Call sendDataThroughUart().

// UART Data Transmission

Function sendDataThroughUart():
Populate the data packet:
- DataPacket[0] and DataPacket[1]: ECG data (ecgsigned16).
- DataPacket[2] and DataPacket[3]: IR data (PPG, irunsigned16).
- DataPacket[4] and DataPacket[5]: filtered SBKP signal (SBKP 16).
- DataPacket[6] and DataPacket[7]: PTT (intervaltime6).
Send the packet header through UART.
Send the sensor data through UART.
Send the packet footer through UART.

Additional note: For the source codes of processing 4.0 (Java), and Bluetooth connection, please refer to the experimental section.
